# Emulating interactions between microorganisms and tumor microenvironment to develop cancer theranostics

**DOI:** 10.7150/thno.70719

**Published:** 2022-03-14

**Authors:** Tongmeng Jiang, Tao Yang, Yingfan Chen, Yao Miao, Yajing Xu, Honglin Jiang, Mingying Yang, Chuanbin Mao

**Affiliations:** 1 School of Materials Science and Engineering, Zhejiang University, Hangzhou, Zhejiang 310027, P. R. China; 2 Institute of Applied Bioresource Research, College of Animal Science, Zhejiang University, Yuhangtang Road 866, Hangzhou, Zhejiang 310058, P. R. China; 3 Department of Chemistry and Biochemistry, University of Oklahoma, Norman, OK 73019, USA

**Keywords:** microorganisms, tumor microenvironment (TME), cancer theranostics, microbiota spectra

## Abstract

The occurrence of microorganisms has been confirmed in the tumor microenvironment (TME) of many different organs. Microorganisms (e.g., phage, virus, bacteria, fungi, and protozoa) present in TME modulate TME to inhibit or promote tumor growth in species-dependent manners due to the special physiological and pathological features of each microorganism. Such microorganism-TME interactions have recently been emulated to turn microorganisms into powerful cancer theranostic agents. To facilitate scientists to explore microorganisms-TME interactions further to develop improved cancer theranostics, here we critically review the characteristics of different microorganisms that can be found in TME, their interactions with TME, and their current applications in cancer diagnosis and therapy. Clinical trials of using microorganisms for cancer theranostics are also summarized and discussed. Moreover, the emerging technology of whole-metagenome sequencing that can be employed to precisely determine microbiota spectra is described. Such technology enables scientists to gain an in-depth understanding of the species and distributions of microorganisms in TME. Therefore, scientists now have new tools to identify microorganisms (either naturally present in or introduced into TME) that can be used as effective probes, monitors, vaccines, or drugs for potentially advancing cancer theranostics to clinical applications.

## 1. Introduction

Cancer has been reported as one of the leading causes of death and a monster that tortures the quality of life worldwide in the 21^st^ century 1. Urgency has been coming out since its diagnosis and therapy remain challenging. For centuries, the knowledge of tumor microenvironment (TME) stands for the interaction of cancer cells and the milieu networks around them, providing insights for understanding how the heterogeneous cells generate, proliferate, migrate, develop and even invade or contaminate normal cells in nature 2. Recently, numerous studies have demonstrated that microorganisms play pivotal roles in forming and changing TME and developing cancer theranostics 3.

Microorganisms include all kinds of microbiota such as bacteria, viruses, phages, protozoa, and fungi 4. Great evidence has been presented to highlight the impacts of microorganisms in physiological and pathological features, such as metabolism, inflammation, and immunity 5. Traditional ideas mainly focus on the dysbiosis of microorganisms and their nosogenetic impacts of inducing a variety of diseases, including but not restricted to rheumatoid arthritis 6, HIV 7, Parkinson's disease 8, liver cirrhosis 9, inflammatory bowel disease 10, graft-versus-host disease 11, type 2 diabetes mellitus 12 and different kinds of cancer 13. However, the advantages of microorganisms have been ignited up by recent findings and current strategies. Symbiotic microorganisms live commensally in bodies and modulate health and development from prenatal to postnatal periods through microbiota-host interactions 14. There are trillions of commensal microorganisms naturally existing in skin 15, lung 16, oral cavity 17, esophageal 18, stomach 19, gut 20, vagina 21 and etc. Battle with pathogenic microorganisms maintains homeostasis and regulates overall health. Moreover, a new epoch has been in advent due to the development of incumbent technologies based on *in vivo* imaging 22, CRISPR/Cas 9 23, phage display 24 and immunotherapy 25, leading to more convenient and effective prognostics, diagnosis and therapies for various diseases, especially cancer.

Over the past several decades, a new approach, theranostics, referring to diagnostics and therapy, sparked the prosperity of cancer treatment with high accuracy and specificity owing to the development of nanomedicine 26. Conventional theranostic platforms using inorganic nanoparticles such as iron oxide nanoparticles (IONP), gold-based nanoparticles and quantum dots (QD) present great potential advantages and seem to reach the clinical translation status 26. However, they leave some drawbacks such as low biocompatibility, high toxicity, non-biodegradability and lack of targeting 26. Hence, biological obstacles, including enzymatic substrates, naturally-derived transporters, microorganisms, and cells, were applied to overcome the former blemishes and further improved the next generation of cancer theranostics 26. Among these biological obstacles, microorganisms are known for their easy applications in the area of cancer theranostics by serving as probes 27, monitors 28, drugs 29 or immunotherapeutic composites 30. More importantly, the microorganisms per se provide unique structures and characteristics that make themselves beneficial for cancer theranostics. For example, the head of T4 phage contains immunomodulators 31 that can be exploited for cancer therapy 32. Oncolytic vesicular stomatitis virus (VSV) stimulates the innate immune system and proinflammatory responses, thus inhibiting melanoma 33. Also, as an immunomodulator, *Listeria monocytogenes* may induce bacteria for stimulating CD8+ cytotoxic T-cells that are cancer killers 34. *Ganoderma sinense* inhibits H1299 non-small-cell lung cancer *ex vivo* and* in vivo* 35 mainly because it contains polysaccharides that can regulate immune cells and induce cytokines 25. Application of *Trypanosoma cruzi* epimastigotes aggrandizes the NADPH oxidase activity to inhibit tumorigenesis because it systematically activates macrophages, dendritic cells, CD4+ and CD8+ T cells 36. Therefore, microorganisms are promising next-generation theranostic platforms.

**Figure 1:**
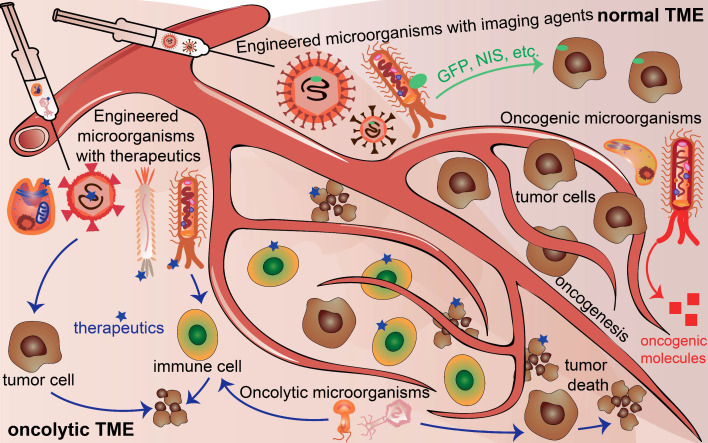
Microorganisms existing in normal tumor microenvironment (TME) and oncolytic TME present different functions. Oncogenic microorganism expressed molecules that stimulate oncogenesis. Engineered microorganisms are designed as monitors or diagnostic factors in normal TME, and serve as monitors or therapeutic factors in oncolytic TME. Some microorganisms are acting as anti-tumoral therapeutics themselves in TME.

However, there are still challenges for using microorganisms in the field of cancer theranostics because of the complicated interactions between cancer cells and microorganisms. First, the distinctive structures and properties of different microorganisms exert distinguished contributions to different cancers. Second, even the same microorganisms affect cancer cell proliferation, progression, and death discrepantly at different stages or time points of the tumor development for the same type of cancer. Third, cancer cells excrete growth factors and molecules, thereby influencing the survival and functions of the microorganisms. Moreover, in normal TME, oncogenic microbiota induce oncogenesis, beneficial microbiota suppresses oncogenesis, and engineered microbiota injected into normal TME could serve as tumor monitors or diagnostic factors. In contrast, engineered microbiota injected into TME could not only provide monitor functions but also act as therapeutic factors, developing TME into an oncolytic milieu **(Figure 1)**. This review explores the basic knowledge of the characteristics of microorganisms, their interaction with cancer, and their potential applications for cancer theranostics.

## 2. Structures and properties of microorganisms used in cancer theranostics

### 2.1. Bacterial viruses (phages)

**Figure 2:**
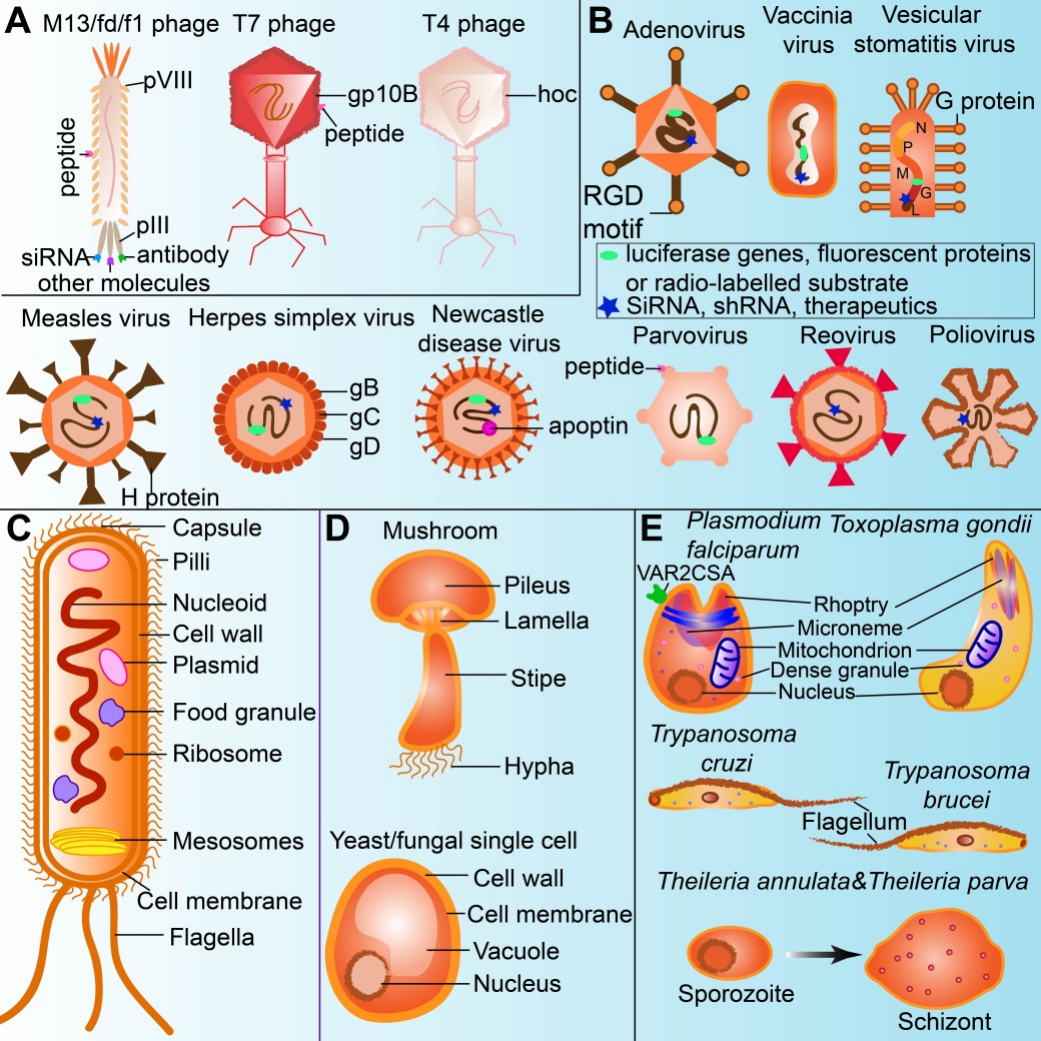
The unique structures of microorganisms applied for cancer theranostics. (A) Phage can be modified for displaying tumor-targeting peptides, anti-tumor agents, as well as acting as oncolytic factors themselves. (B) Oncolytic viruses can be labelled with imaging agents or therapeutics for cancer theranostics. (C) Bacteria uptake nanoparticles or imaging agents as food granule for tumor imaging. Bacterial mesosomes and ribosome contribute to excrete anti-cancerous enzymes or agents. The flagella and LPS in the bacteria cell wall can modulate immune response for cancer therapy. (D) Polysaccharides in the cell walls of fungi (including mushroom and yeast) could stimulate immune cells for cancer therapy. (E) Protozoa expressing or modified with therapeutics (ie.VAR2CSA) and flagella in the protozoa are sometimes anti-tumorous. Transformation of sporozoites into schizonts stimulate apoptosis and proinflammation.

In general, bacterial viruses, which are referred to as bacteriophages or phages, are verified to bear capacities in infecting bacteria. They cannot replicate without host cells in nature 37. Phages are generally viruses containing single or double-stranded nucleic acids (DNA or RNA) that are protected by proteins with or without tails. They are principally classified into two categories, including lytic phages and lysogenic phages, depending on the replication status when they are formed in the host cells 38. Basically, phages range from 24 to 400 nm in size 39, and consist of the capsid (head), which protects the genetic materials with or without tails and other exceptions 40, 41. Lytic phages are named by their lytic cycle, whereas lysogenic phages only follow the lysogenic cycle 42. Genetic materials of the phages are integrated into and replicated along with the host cells at the lysogenic cycle 43, followed by the lytic cycle if activated 43. Lytic phages used the host biosynthetic machines to produce the genetic materials, coated with proteins and lysis proteins before mature phages appear. Then the mature phages get the host cells ruptured since enough lysis proteins accumulated 42, 44. Filamentous phages such as M13, fd, and f1 are lysogenic and replicate without killing the host cells. They are thus usually used in phage display technology to express peptides or antibodies, especially on pIII and pVIII proteins 45. Unlike filamentous phages, T7 phage presents proteins or peptides on the capsid protein gp10B 46. In addition, the highly immunogenic outer capsid protein (gene product hoc) in phages can modulate the immune response; especially, the gene product hoc in T4 phage head contains immunoglobulin superfamilies 31. Therefore, they are candidates for cancer therapy 32 **(Figure 2A)**.

### 2.2. Oncolytic viruses

Viruses other than bacterial viruses, especially oncolytic viruses, are the ones that could exist in tumors for cancer theranostics. Oncolytic viruses are tumor-selective replicating tools, which can effectively kill tumor cells with acceptable side effects on normal cells 47. Several viruses have exhibited their oncolytic characteristics, such as adenovirus, vesicular stomatitis virus (VSV), vaccinia virus, reovirus, and herpes simplex virus (HSV) because of their unique structures 47. For example, an adenovirus contains episomal dsDNA ranging from 30kb to 38kb and is coated with a capsid mostly carrying RDG motifs. It could infect a large number of cells with integrins or coxsackievirus and adenovirus receptor (CAR) no matter whether they are dividing or not 47. Likewise, the G protein on the VSV surfaces infects many tumorous cells 48, the H protein on the spike of measles virus recognizes CD46 or signaling lymphocyte activation molecule (SLAM) on mammal cells 49, and the envelope glycoproteins (gB&gC) on HSV interact with the surface heparin sulfated proteoglycans (HSPGs) on mammal cells to further help glycoproteins (gD) stimulate nectin-1 or herpes viral entry mediators (HVEM) on mammal cells 50. Thus, they provide enough biological access for genetic editing and gene modification. Especially, they can be armed with luciferase genes, fluorescent proteins or radio-labelled substrate molecules for tumor imaging 51 as well as siRNA, shRNA or therapeutics for tumor inhibiting 52. For example, engineered Newcastle disease virus with apoptin could activate tumor death 53 **(Figure 2B)**. Oncolytic viruses are also activators of toll-like receptor signaling pathways, which induce the acute inflammatory reactions of local tumors 33.

### 2.3. Bacteria

Bacteria are single-cell microorganisms basically consisting of cell walls, cell membranes, cytoplasm, nuclear bodies, and other spatial structures, including capsule, flagellum, fimbria, and endospore. Bacteria were used as an anti-cancer agent by German physicians one hundred and fifty years ago. Since then, they have been found useful in cancer therapy. Tumors regressed when they were infected by certain kinds of bacteria, such as *Streptococcus pyogenes* for neck cancer, Bacillus Calmette-Guérin (BCG) for bladder cancer, and *Clostridium histolyticum* for metastatic cancer 54, 55. Some bacteria are naturally existing to form colonization and inherent to tumors, and thus they can excrete anti-cancerous enzymes or agents by mesosomes and ribosomes. More specifically, anaerobic bacteria can easily survive in the TME with underprivileged oxygens, but anaerobic bacteria could destroy the tumor. Gram-negative anaerobes, such as *Salmonella* could get into the tumors and grow both inside and outside, while Gram-positive anaerobes, such as *Clostridia* and *Bifidobacteria*, proliferate in the TME without or with oxygen even in the presence of tumor necrosis 54. Owing to the simple genomes of bacteria, they could be genetically engineered as vectors to carry and deliver various anti-cancerous agents, including but not limited to siRNA, shRNA, microRNA, therapeutic DNA, immunomodulators, antiangiogenic and cytotoxic molecules 56. Bacteria that could uptake nanoparticles or imaging agents (i.e. ^18^F-FDS) as food granules could be employed in tumor monitoring and imaging 57. The flagella and LPS in the cell wall are mediators for different immune cells, including CD8+ T cells, Treg cells, macrophage, NK cell and dendritic cells in TME 58 **(Figure 2C)**.

### 2.4. Fungi

Fungi are eukaryotic organisms, which can attack, infect or influence the human body under diverse circumstances. Cell walls are critical components of fungi (including mushroom and yeast), which help assist fungi in resisting environmental stress and invading ecological niches 59. Polysaccharides make up more than 90% of fungi cell walls with extension decorations determined by the pathogens 60, which are currently called “pathogen-associated molecular patterns” (PAMPs), including β-glucan, mannans, and chitin 61 **(Figure 2D)**. Human bodies recognize PAMPs by innate immune cells through cascade signaling pathways of pro-inflammatory and anti-inflammatory cytokines, such as retinoic acid-inducible gene 1 (RIG-I)-like receptors (RLRs), nucleotide oligomerization domain (NOD)-like receptors (NLRs), Toll-like receptors (TLRs) and C-type lectin receptors (CLRs) 62. Pro-inflammatory cytokines are often key factors inducing oncogenesis 63, whereas anti-inflammatory cytokines may support cancer therapy 64.

### 2.5. Protozoa

Protozoa are eukaryotic organisms dwelling in extracellular fluids or inside host cells due to their innate evasion and resistance to the human immune system 65. There are several strategies for protozoa to get away from humoral immune defenses so that they could further affect human bodies. Firstly, an isomeric host complement-mediated compound named 160-kD glycoprotein (gp160) is expressed to conjugate C3b and C4b and then suppress the complement-mediated lysis of protozoa 66, 67. Some protozoa such as *Leishmania* have modified surface lipophosphoglycan (LPG), which acts as a barrier to protect parasites from being attacked by lytic C5b-C9 membrane attack complex (MAC) 68. Similarly, other protozoa like *Trypanosoma brucei* resist primate-specific trypanosome lysis factors (TLFs) based cytotoxicity due to their well-known structure called flagellar pocket 69. *Trypanosoma cruzi* may present similar effects on the mammalian cells due to its same flagellum structure 70. *Plasmodium falciparum* expressed VAR2CSA that could target tumors for cancer theranostics 71. Secondly, protozoa remodel the compartments of host cells and inhibit host cell signaling pathways that contribute to antimicrobial mechanisms 65. *Toxoplasma gondii* restricts the fusion of lysosomes and endosomes by dwelling in phagosomes, whereas *T. cruzi* destroys the Ca^2+^-regulated lysosomal exocytic pathway in mammalian cells 72. Thirdly, some protozoa (i.e., *Plasmodium falciparum*) impair the capacity of dendritic cells (DCs) to activate antigen-specific primary and secondary T cell responses by binding to myeloid DCs 73. *Plasmodium falciparum* exerts some organelles (i.e., rhoptry, microneme and dense granule) just like *Toxoplasma gondii*, therefore, both may display some similar properties in the TME. Sporozoites of *Theileria annulate* and *Theileria parva* transformed into schizonts in mammalian leucocytes, meanwhile stimulating apoptotic and proinflammatory effects 74 **(Figure 2E)**.

## 3. Interactions between microorganisms and tumors

Normally, microorganisms influence the homeostasis of the host. Some microbial communities reside in the oral, skin, gut, nasal cavity, lung, pancreas, prostate, urinary or genital tract and coexist with the human body peacefully. In contrast, others stimulate chronic immune reactions or even enter tumors. For one thing, pathogenic microorganisms contribute to disease development by excreting metabolites, stimulating an immune response, and activating inflammatory pathways. For another, commensal microorganisms residing at the barrier sites exert protecting effects through resisting pathogens and regulating the immune response and metabolism of the host, such as inducing migration of immune cells, stimulating chemokines and cytokines etc 13. In the TME, the relationship between microorganisms and tumors depends on the place of tumor occurrence as well as the category of microorganisms. For decades, some TMEs, in particular in the lung, have long been demonstrated as a sterile environment by oncologists. However, this hypothesis has been challenged by the current technologies and recent studies 75. There is increasing evidence that microorganisms are naturally present in tumors or around tumors, constitute TME, and participate in tumor development. In addition, some microorganisms restrict the development of tumors on the one hand, and some others contribute to tumor growth on the other hand.

### 3.1. Microorganisms naturally present in tumors of different organs

#### 3.1.1. Respiratory tract

Many microbiotas reside in the upper and lower respiratory tract from the nasal cavity, pharynx, larynx to the trachea, bronchi, and lung. Among them, several microorganisms are living with respiratory tumors. Significant different diversities of bacterial microbiomes have been detected using bacterial 16S rRNA sequencing between the normal nasal cavity and malignant nasal neoplasia 76. Gong et al. compared the profiles of microbiotas among normal larynx, laryngeal cancer, and the normal tissues adjacent to laryngeal cancer and found the different populations of microorganisms among them 77. Evidence has also shown the discrepancies of microorganisms in other lung cancer tissues by biopsy or bronchoscopy 78.

#### 3.1.2. Oral-gut axis

The digestive tract begins from the oral cavity to the anus throughout from the outside of the body to the inside of the body as well as the appendicle organs, including the liver, gallbladder, and pancreas. Thus a majority of microorganisms live in the digestive tract. Since it is a long tract of tubes with complexities of structures and circumstances, there are multiple factors to generate divers of tumors by microbiome residents, including pathogenic, opportunistic, and commensal microorganisms. Different from the infective and opportunistic microbiota in the digestive tract leading to cancer, the commensal microbiomes could promote the body's health and prevent cancer 79. To date, the data of commensal microbiomes in the digestive tract has been well established, especially in the gut.

#### 3.1.3. Urinary-genital axis

Similar to the gastrointestinal tract, the genitourinary organs are also easy to form tumors and have abundant commensal microorganisms because the sterile environment of the genitourinary tract has been abandoned 80. In an interesting way, the *Actinomycetes* and BCG around and in bladder cancer prevent tumor relapses and show potential treatment effects for bladder cancer 81. For females, HPV-induced cervical carcinogenesis has been demonstrated to be linked with microbiota dysbiosis in the vagina/cervix and cervical cancer 82. Not coming singly but in pairs, for males, Bacteroides and Streptococcus species have been detected in prostate cancer. However, their roles need further exploration 83.

### 3.2. Microorganisms naturally present in TME

Once upon a time, the infection of microbiomes, including phages, viruses, bacteria, fungi, protozoa etc. has been concerned because it is thought to result in tumorigenesis and carcinogenesis not only *in situ* but also in distant tissues or organs. Pathogenic microorganisms play multiple oncogenic roles that contribute to cancer formation and development. A wide range of microorganisms can get through the human body from skin, mouth, and other trenches like wounds. There are a large number of studies reporting the relationship of pathogenic viruses and tumors, such as Epstein-Barr virus (EBV) for nasopharyngeal carcinoma 84, hepatitis virus for liver cancer 85, human papillomavirus (HPV) for oropharyngeal 86 and cervical cancer 87, and human T-lymphotropic virus for leukemia 88. Likewise, bacteria are also the sinful archcriminal in oncogenesis. For example, *Helicobacter pylori (H. pylori)* contributes to gastric cancer 89, *Bacteroidetes*, *Verrucomicrobia*, and *Proteobacteria* to gut cancer 90, and *Veillonella*, *Megasphaera* to lung cancer 91. What is more, fungi and protozoa are also criminals that promote the initiation and development of cancer. For example, *Aspergillus flavus* lead to liver cancer 92, and *Liver fluke Clonorchis sinensis* causes cholangiocarcinoma 93. Besides, microorganisms naturally present in TME sometimes play an anti-tumor role. This phenomenon was mostly found in phages and bacteria. In this section, we discussed the oncogenic roles (phages, viruses, bacteria, fungi, and protozoa) and the anti-tumorous roles (phages and bacteria) of microorganisms naturally in TME **(Table 1-3)**.

#### 3.2.1. Bacterial viruses (phages)

As microorganisms naturally present in the environment, phages are also present in the human body and TME 94. Firstly, phages are suspected to bind integrin proteins (e.g. αIIbβ3, αvβ3), which are expressed on tumorous cells and activated T cells 95. Secondly, phages can mediate invading pathogens that are tumor inducers 96, and thus may suppress tumor growth. In particular, endogenous phages modulate bacteria in the oral-gut axis, therefore maintaining the microbiota homeostasis in TME 96. Third, phages can stimulate different immune cells, which are important cells in TME. For example, T4 phages activate dendritic cells 97, inhibit CD3 receptor-induced T-cell proliferation, and stimulate the migration of granulocytes and mononuclear cells 98. In addition, phages control the homeostasis of host immune reactions in tumor-bearing animals and humans, therefore influencing TME 99. Some metagenomic analysis presents certain type of phages related to TME 96, 100, and those residing in TME 96 are listed in **Table 1**.

#### 3.2.2. Other viruses

Viruses naturally present in TME are oncogenic factors. Some viruses express oncogenes which induce tumorigenesis by influencing cell cycles and DNA damage processes 101. For instance, E6 and E7 expressed by HPV induce anal cancer, cervical cancer, and vaginal cancer; LANA and v-cyclin expressed by Kaposi's sarcoma herpes virus (KSHV) induce Kaposi's sarcoma and primary effusion lymphoma; NS3, NS4B, NS5A and core proteins expressed by hepatitis C virus (HCV) induce hepatocellular carcinoma; HBsAg and HBx expressed by hepatitis B virus (HBV) induce hepatocellular carcinoma; EBNA-1, EBNA-2 LMP-1, and LMP-2 expressed by Epstein-Barr virus (EBV) induce Burkitt's lymphoma, nasopharyngeal cancer, Hodgkin and non-Hodgkin's lymphoma, etc 102. We summarized these viruses in **Table 2**. In particular, the therapeutic approaches for those oncogenic are also included in this table. Moreover, the above oncogenes can also target tumors by binding molecules on the tumorous cells 102. Besides, viruses stimulate oncogenic inflammation by mediating STAT3, MAPK, and NFκB and signaling pathways 103. Also, viruses induce cancers by causing tissue injury. For example, HBV and HCV trigger liver cirrhosis and hepatocarcinogenesis 104. Additionally, some viruses promote tumor growth and progression by modulating cytokine/chemokine networks 105 and manipulating cell cycles and DNA damage processes 101.

#### 3.2.3. Bacteria

Similar to viruses, bacteria are also modulators of inflammation and induce oncogenesis 106. For example, FadA molecules expressed by *Fusobacterium nucleatum* regulate the inflammation and oncogenesis in colorectal cancer due to its binding to E-cadherin and activation of β-catenin signalling 107. In addition, F. nucleatum also plays tumorigenic roles in inhibiting T cell proliferation and inducing T cell apoptosis in colorectal cancer 108. Bacteria also produce carcinogens such as bile acids, H_2_S and deoxycholic acid 109. In contrast to the oncogenic effects of bacteria, they also exert important anti-cancer effects by modulating the cytokine/chemokine networks and immune cells in TME 34, especially for some commensal bacteria in colon cancer 110 **(Table 3)**.

#### 3.2.4. Fungi

The roles of fungi naturally existing in TME are also oncogenic. They produce carcinogens such as nitrosamines and acetaldehyde 109. Glycans as major components of fungal walls trigger complement cascade in TME 111. Another mechanism for fungi to promote cancer is molecular mimicry 109. For instance, *Candida albicans* expresses complement receptor 3-related protein (CR3-RP), which has a similar structure to CR3 on the leukocytes, interfering with the immune response in TME 109 **(Table 3)**.

#### 3.2.5. Protozoa

Traditionally, protozoa are parasites not only known as pathogenic factors but also play tumorigenic roles. The oncogenic roles of protozoa are mainly manifested in stimulating inflammation, modulating cytokine/chemokine networks, and triggering the response of immune cells 112. For instance, the interleukin-12 triggered by *Toxoplasma gondii* stimulates T cells and natural killer (NK) cells in promoting cancer 112 **(Table 3)**. Recently, studies show that the functions of microorganisms introduced into TME play anti-tumorous roles, which we are discussed in the next section.

## 4. Applications of microorganisms used in cancer theranostics

In terms of theranostics, the potential applications of microorganisms for cancer have been well established with the development of nanotechnology. Over the past decades, phage display and other microbiome-based carriers have played a major role in cancer theranostics not only by catering molecules or drugs directly to tumors but also by allowing the visualization or detection of cancer. In this part, we review different microbiota used for cancer diagnosis and therapy** (Table 4-8)**.

### 4.1. Bacterial viruses (phages)

**Figure 3:**
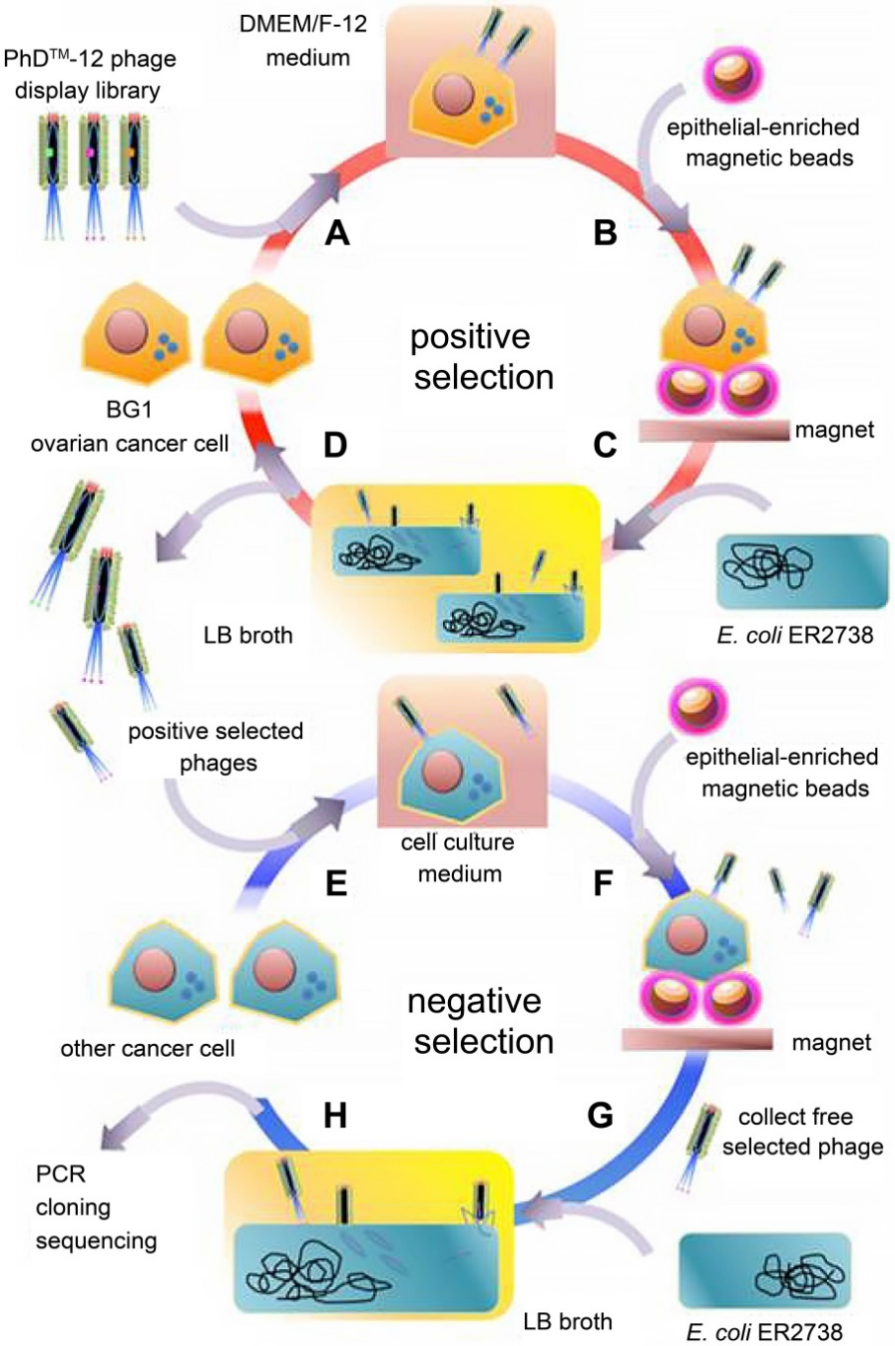
Oligopeptides screened by phage display can be used for ovarian cancer diagnosis. Adapted with permission from 116, Copyright 2015, Ivyspring International Publisher, CC BY-NC 4.0.

**Figure 4:**
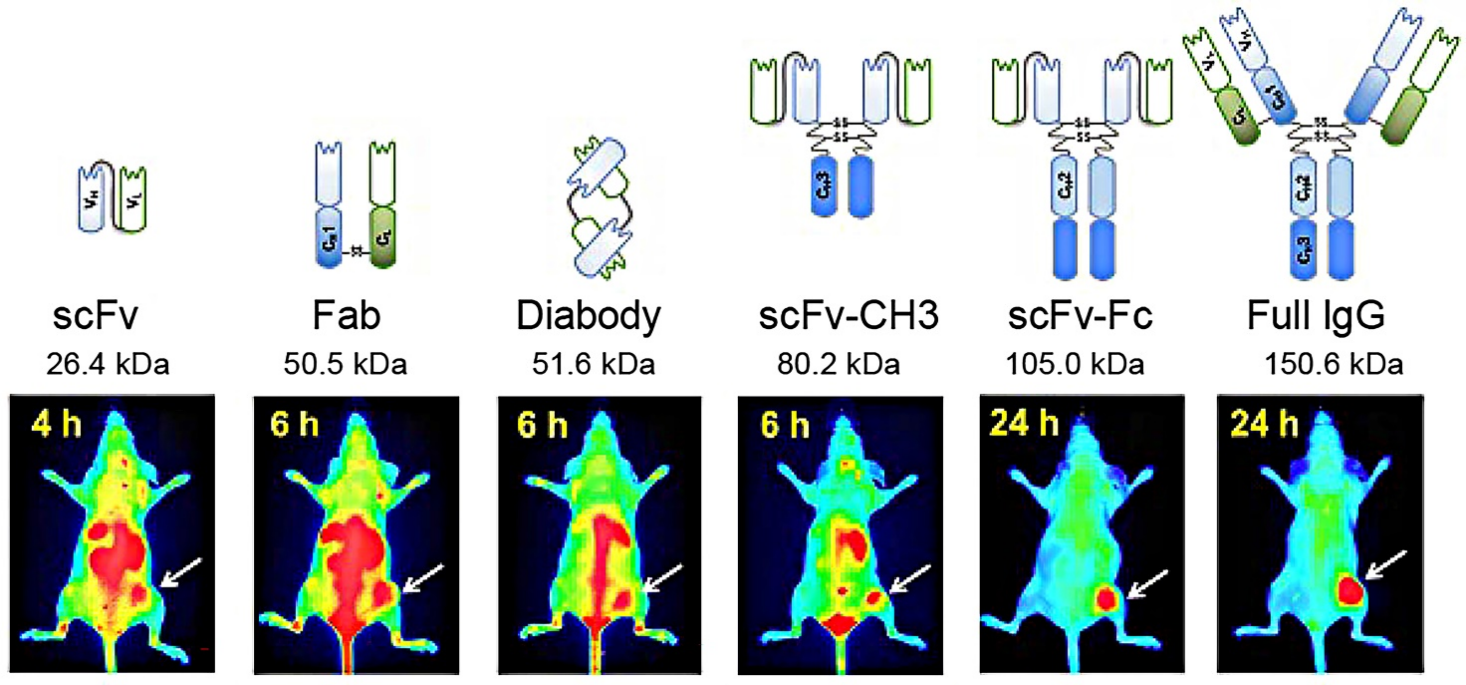
Near-infrared fluorescence imaging probes based on M13KO7 phage display. Adapted with permission from 117, Copyright 2018, Ivyspring International Publisher, CC BY-NC 4.0.

**Figure 5:**
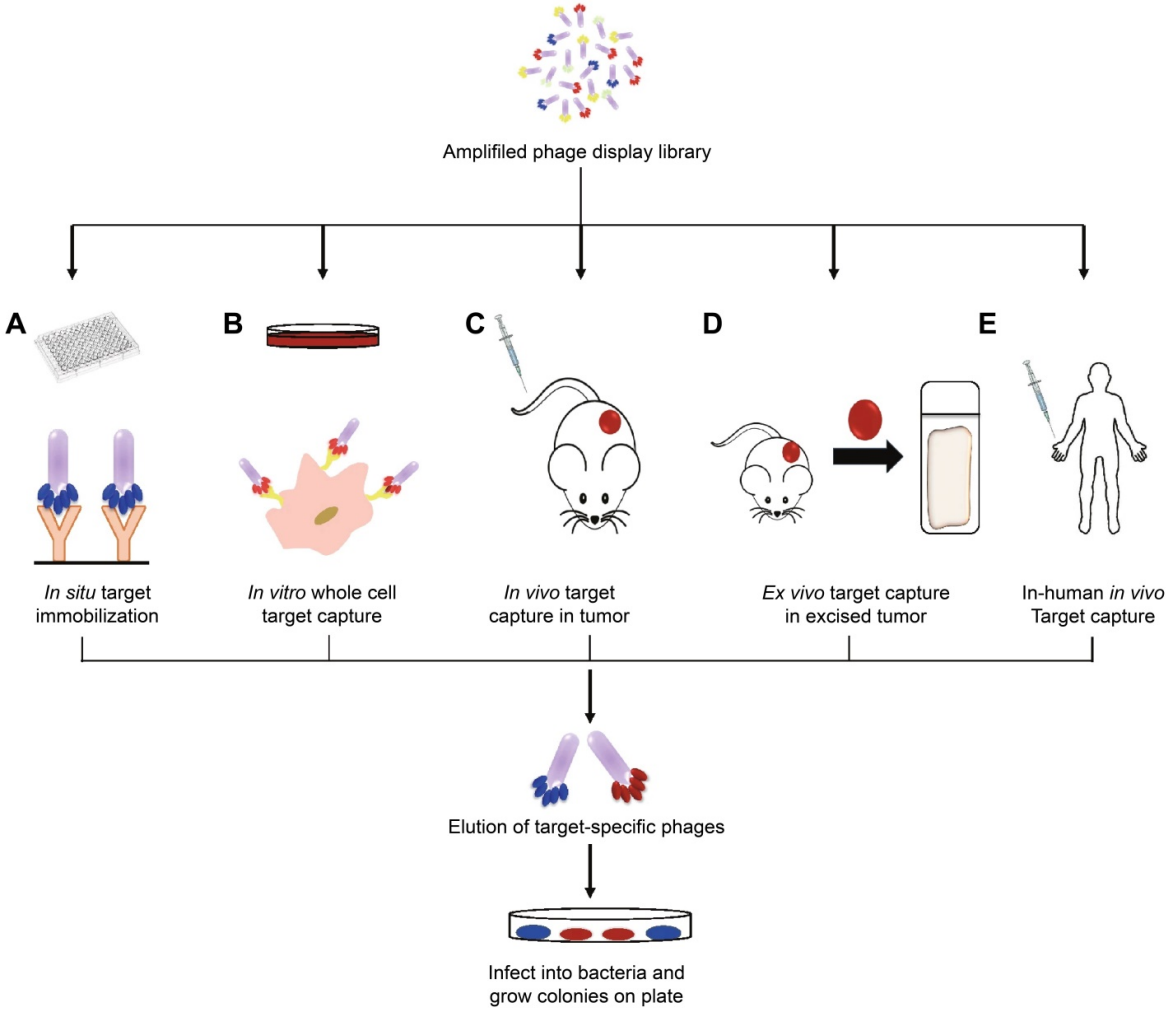
Peptides screened by phage display and used for targeted cancer therapy. Adapted with permission from 119, Copyright 2019, Springer Nature Switzerland AG. Part of Springer Nature, CC BY 4.0.

The boost of phage display opens a new era for cancer theranostics, especially since this technology was awarded Nobel Prize in 2018 113. Phage display can be used to visualize cancer location and further reflect the behaviors and activities of cancer 114. Phage display technology has contributed to cancer theranostics in the following aspects **(Table 4)**. First, phage antibody library screening is used for selecting accurate targets for detecting cancer at the early stage. Second, phage display-derived peptides are utilized as imaging probes for monitoring cancer. Third, phages containing nanoparticles or small molecules as drugs could help prognosticate cancer. For instance, a phage-displayed random peptide library can be used to identify the epitope sequences, such as pinpointing CSPG4 as a target for theranostics of B-cell lymphoma 115. Likewise, integration of an M13mp19 phage-displayed peptide library and a microfluidic system discovered cancer cell-specific oligopeptides for ovarian cancer diagnosis **(Figure 3)** 116. AF680-labeled phage nanoparticles with targeting peptides are utilized for ovarian cancer cell line imaging by fluorescent microscopy 22. M13KO7 phage display was employed to isolate an anti-HER3 antigen-binding fragment as a near-infrared fluorescence imaging probe for imaging HER3-positive cancer through positron emission tomography (PET) **(Figure 4)** 117. In addition, M13 phage based probe is a powerful method for the detection of circulating tumor cells 118. Besides, peptides screened by M13mp19 phage display can also be applied for targeted cancer therapy by targeting the TME, receptors on cancerous cells, or tumor vasculature **(Figure 5)** 119.

Except for M13 phage, T4 and T7 phage display has also been employed for identifying tumorous antigens, screening targeting peptides, and generating vaccines for cancer theranostics 32, 46. For example, we generated a naked eye counting system to detect the cancer-biomarker miRNAs by fluorescent T7 phage 120. Besides, fd phage is applied for cancer diagnosis and therapy. For instance, we increased the detection sensitivity of anti-p53 antibody, a cancer biomarker, by a combination of antigens and fd phage nanofibers 121. Our group has also developed antiangiogenic targeted breast cancer therapy based on angiogenin-binding peptides displayed on the side wall of fd phage as well as the tumor-homing peptides displayed at the tip of the same phage 122. Moreover, coat proteins derived from fd-tet phages could guide the delivery of small interfering RNA (siRNA), leading to efficient breast cancer gene therapy 123. Many clinical trials of monoclonal antibodies based on phage display have been launched for cancer chemotherapy 124, such as Mapatumumab for lymphoma 125, colorectal cancer 126, and Drozitumab for chondrosarcoma, ovarian and colorectal cancers 127.

### 4.2. Oncolytic viruses

**Figure 6:**
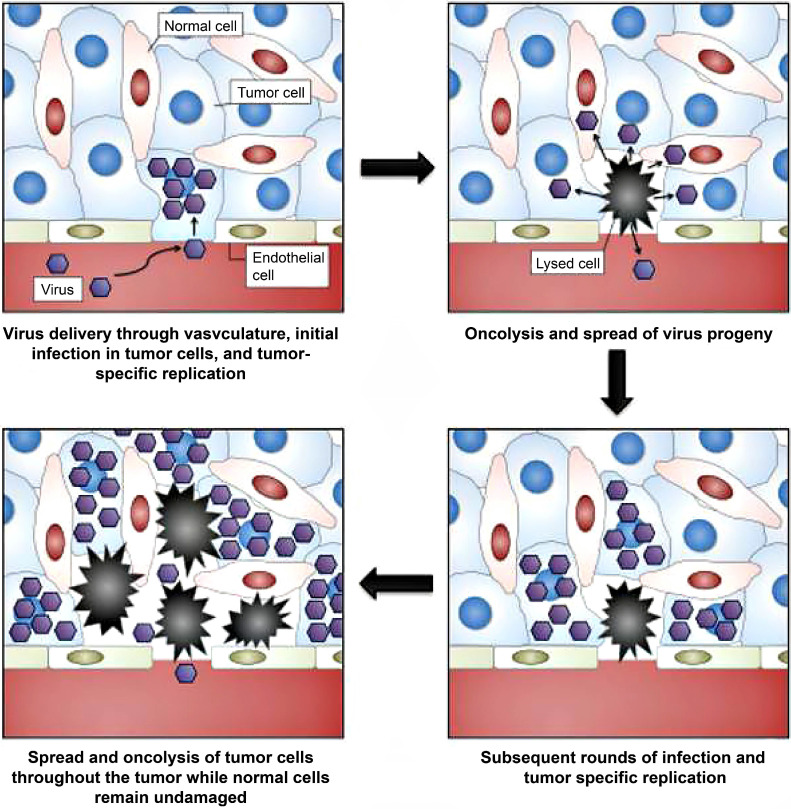
Selection of oncolytic vaccinia virus for personalized therapy. Adapted with permission from 51. Copyright 2012, Ivyspring International Publisher, CC BY-NC 4.0.

**Figure 7:**
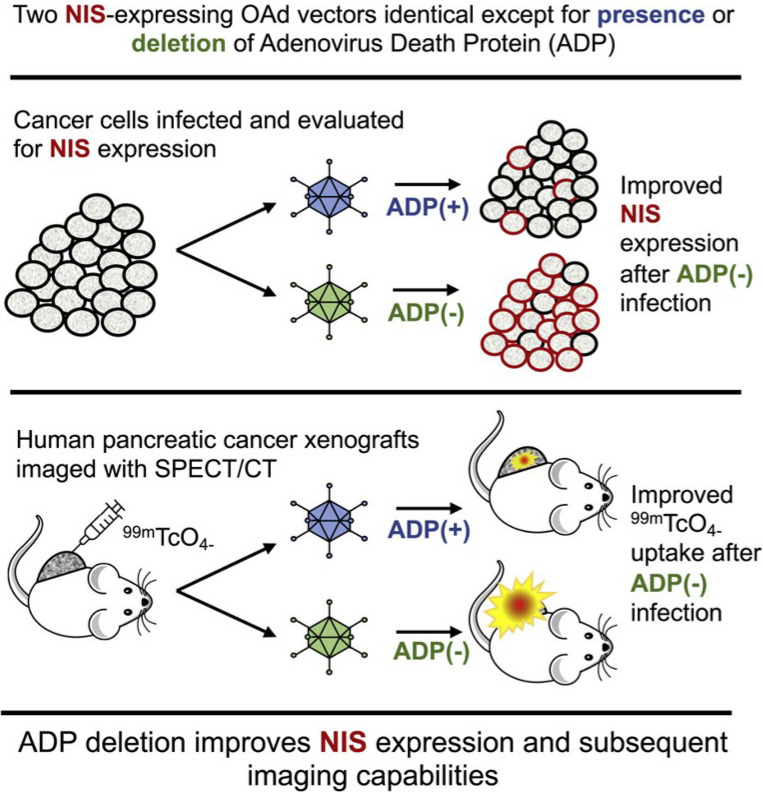
Oncolytic NIS-expressing adenovirus enhances cancer imaging in pancreatic cancer models. Adapted with permission from 135, Copyright 2021, Elsevier, CC BY-NC-ND 4.0.

**Figure 8:**
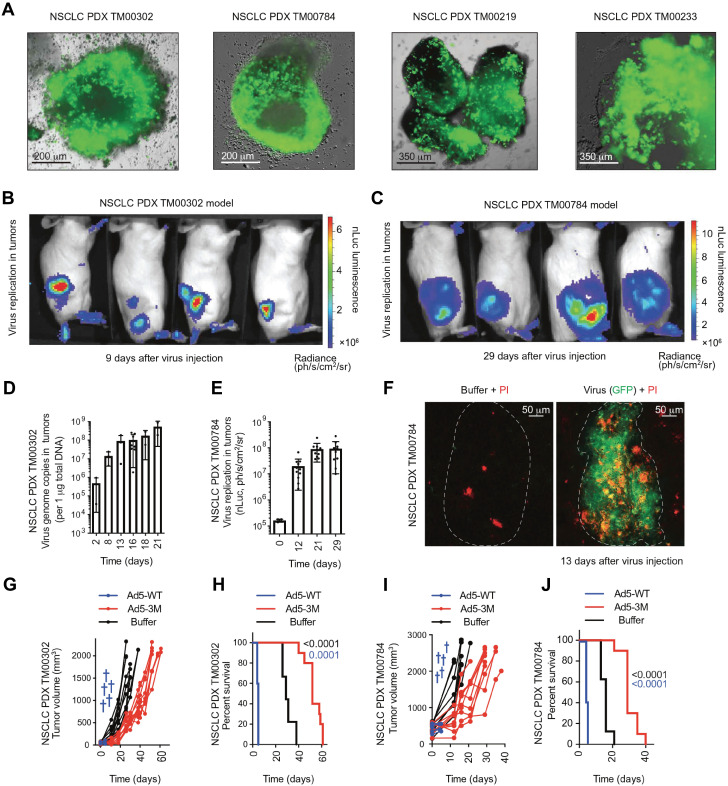
Administration of engineered adenoviruses suppresses tumor growth and prolongs survival of lung cancer bearing mice. (A) Bioluminescence images of subcutaneous tumor-bearing mice after administration of viruses. (B) Activity of viruses. (C) Amounts of viruses. (D) Tumor volume after administration of viruses. (E) Survival of subcutaneous tumor-bearing mice after administration of viruses. (F) Viral genome copies in the lungs after administration of viruses. (G) Immunofluorescent staining of lung tumors after administration of viruses. (H) Survival of orthotopic tumor-bearing mice after administration of viruses. (I) Bioluminescence images of orthotopic tumor-bearing mice after administration of viruses. (J) HE staining of lung tumor. Adapted with permission from 143, Copyright 2020, The American Association for the Advancement of Science.

Unlike phages, oncolytic viruses are utilized for cancer theranostics in a different way **(Table 5)**. Oncolytic viruses are used as anti-cancer vaccines generally in two directions. First, large viruses can cause diseases and rarely replicate in normal tissues. But they are abundant in tumors such as poliovirus 128, herpes simplex virus (HSV) 50, adenovirus 52, and vaccinia virus 129. These viruses bear virulence genes that replicate with tumor proliferation and play roles in anti-proliferation, anti-apoptosis, and immune modulators **(Figure 6)** 51. Second, small viruses have fast replication cycles and normally do not result in diseases, including vesicular stomatitis virus (VSV)^130^ and reovirus 131. These viruses are commonly used as vectors for gene therapy. Compared to large viruses, they are safer carriers for both *in vitro* and *in vivo* cell transfection 132. Besides, many monitoring systems, including bioluminescence imaging, fluorescence imaging, and nuclear medicine-based imaging, are widely applied both experimentally and clinically, which is based on the backbones of oncolytic viruses (Adenovirus 133-135, HSV-1 136, measles virus 137, Newcastle disease virus 53, parvovirus 138, vaccinia virus 139 and VSV 140-142) or the genes armed on them 51. For instance, engineered oncolytic measles virus (MV-GFP-HSNS-scEGFRvIII and MV-GFP-HAA-scEGFRvIII) can not only induce GFP expression for imaging the EGFRvIII-expressing glioma lines and xenografts but also present an antitumor activity 49. Oncolytic adenoviruses not only can be armed with luciferase cDNA 133, green fluorescent protein (GFP) 134, and sodium/iodide symporter (NIS) **(Figure 7)** 135 for tumor imaging but also serve as vectors for the treatment of head-and-neck cancer 52. In addition, engineered adenovirus evades innate immunity *in vivo*, decreases tumor growth, and prolongs survival of lung cancer-bearing mice **(Figure 8)** 143. Nonpathogenic poliovirus triggers antitumor immune responses in TME, treating recurrent glioblastoma in clinical trials 144. Vaccinia viruses not only trigger anti-tumoral immunity by immune cells but also act as vectors for gene therapy for cancers 145, 146.

### 4.3. Bacteria

**Figure 9:**
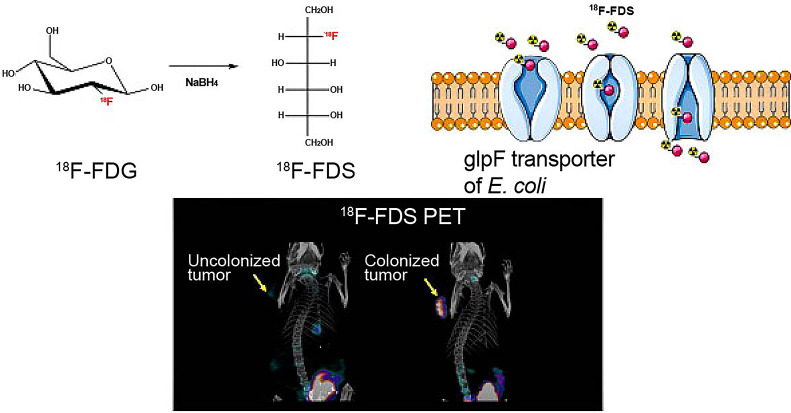
Bacteria uptake ^18^F-FDS for tumor imaging by PET. Adapted with permission from 57, Copyright 2020, Ivyspring International Publisher, CC BY 4.0.

**Figure 10:**
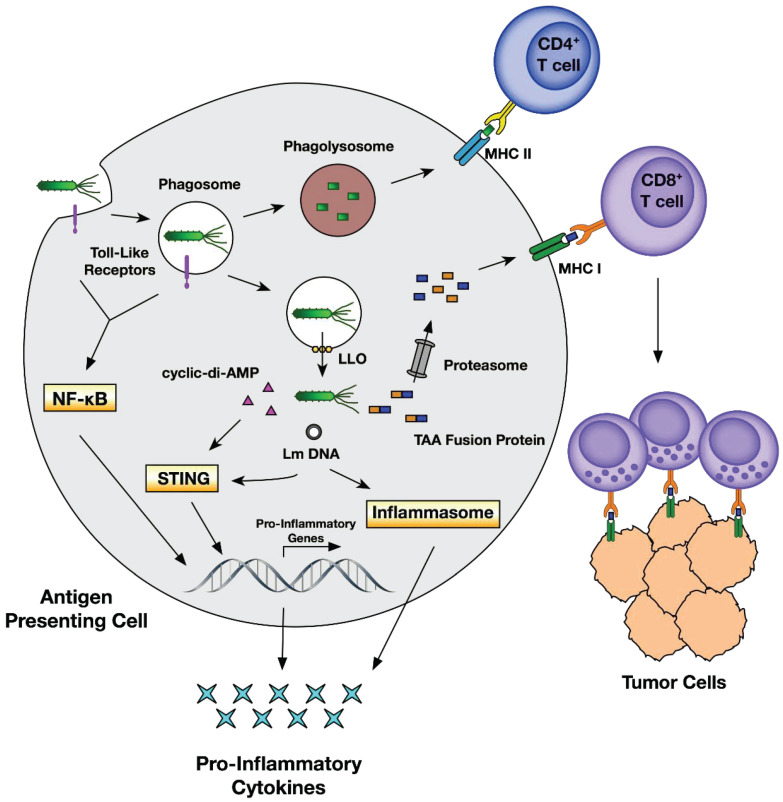
Anticancer effects of Listeria monocytogenes through an immune response. Adapted with permission from 152, Copyright 2018, MDPI, Basel, Switzerland, CC BY 4.0.

Traditionally, bacteria are thought to be deleterious organisms to the human body owing to their pathogenicity that causes different diseases such as infection and cancer 147. Even though bacterial therapy for cancer was claimed as an effective approach a long time ago, it has not been actively studied until the recent findings show their multiple theranostic effects. Briefly, in cancer theranostics, bacteria have been employed as a probe to detect cancer, as a sensor to monitor cancer, and as a therapeutic drug to treat cancer **(Table 6)**. Bacteria-derived elements can also be used as therapeutic drugs for cancer treatment. In addition, bacteria localized to TME modulate chemokines, cytokines, and tumor-infiltrating immune cells, representing a new mechanism by which bacteria target and suppress cancer 148. *Escherichia coli* strain MG1655 injected into tumor-bearing mice can uptake ^18^F-FDS to become visualized by PET imaging of tumors **(Figure 9)** 57. Cytosine deaminase and 5-fluorocytosine derived from *Escherichia coli* inhibit mutant lung cancer A549 cells by activating apoptosis 149. On the one hand, *Salmonella Typhimurium* VNP20009 itself injected into murine melanoma inhibits tumor growth and lung metastasis 150. On the other hand, VNP20009 can also be used as a vector to deliver a specific gene to treat colon cancer in a mice model 151. Likewise,* Listeria monocytogenes* and its products stimulate an immune response (inducing immune cells and modulating cytokines) and act as gene vectors for delivering therapeutics (tumor antigen, DNA plasmid, siRNA, shRNA, etc.) for cancer therapy **(Figure 10)** 34, 152. These gene-targeted therapies are also widely found in *Clostridium sp.*, *Escherichia coli.* and *Salmonella sp.* 153. Mannose-sensitive hemagglutinin armed on *Pseudomonas aeruginosa* inhibits tumor growth and reverses epithelial-mesenchymal transition of skin cancer 154. In addition, many *Listeria monocytogenes* and *Salmonella Typhimurium* strains are employed in cancer therapy 148.

### 4.4. Fungi

Similar to bacteria, fungi have also been found effective in cancer therapy **(Table 7)**. Some compounds derived from medicinal fungi induce mitochondria-mediated apoptosis and thus kill cancer cells 155. These studies investigated the anti-tumor effects of compounds derived from fungi *in vitro*. For instance, polysaccharide-K derived from a mushroom, *Coriolus versicolor*, stimulates apoptosis of leukemia HL-60 cells 156. Ganoderic acids (B, Mf, Mk, S and T) and ribonuclease derived from another mushroom, *Ganoderma lucidum*, also trigger apoptosis in many human cancer cell lines, including colon cancer HCT116 cells 157. Similarly, cordycepin derived from* Cordyceps militaris* (a mushroom) has been used as an anti-tumoral agent in leukemia U937 and NB-4 cells 158 because it can trigger apoptosis and autophagy. Compounds from *Laetiporus sulphureus* present cytotoxic effects on five cancer cell lines, including leukemia HL-60 cells, colorectal carcinoma SW-480 cells, breast cancer MCF-7 cells, lung cancer A-549 cells, and liver cancer SMMC-721 cells 159. Polypeptides from *Pleurotus eryngii* suppress cervical, breast, and stomach cancer cells and modulate macrophages *in vitro* 160. Extracts from *Inonotus obliquus* also inhibit prostatic adenocarcinoma PC-3 cells and breast carcinoma MDA-MB-231 cells 161. Agglutinin from *Paecilomyces japonica* also exerts cytotoxic effects on human breast cancer MDA-MB-231 cells, human pancreas cancer AsPc-1 cells, and stomach cancer SNU-1 cells 162. Pigments derived from Daldinia concentrica 163 and Xylaria schweinitzii 164 also present cytotoxicity against lung carcinoma SK-LU-I cells, hepatocellular carcinoma HepG2 cells, epidermal carcinoma KB cells, and breast carcinoma MCF7 cells. Lectins derived from *Hericium erinaceum* 165, *Russula delica* 166, *Russula lepida* 167, and laccase derived from *Tricholoma mongolicum* 168 can suppress the proliferation of HepG2 hepatoma cells and MCF7 breast cancer cells. Extracts from *Lepista inversa* also suppress cancer cell lines, including NCI-H460 (lung cancer), HCT-15 (colon cancer), AGS (gastric cancer) and MCF-7 (breast cancer) 169. Cytotoxic effects of 5-methylmellein from *Xylaria psidii* 170 and compounds (e.g. cytochalasin, pentaminolarin, xylochalasin, etc.) from *Xylaria sp.* 171 on colon cancer HCT116 cells, prostatic adenocarcinoma PC-3 cells, and MCF7 breast cancer cells are also found to result from the activation of apoptosis. Breast cancer cell lines are also inhibited by orf239342 from *Agaricus bisporus*, Brefeldin A from *Agaricus blazei*, ergosterol from *Amauroderma rude*, organic molecules from *Amauroderma rugosum* 172, culture broth and ethanolic extract from* Antrodia camphorate* 173, extracts from *Clitocybe alexandri* 169, extracts from *Coprinus comatus* 174, extracts from *Flammulina velutipes* 175, ethanol extracts from *Fomes fomentarius* , methanol extracts from *Fuscoporia torulosa*, marmorin from *Hypsizigus marmoreus*, Panepoxydone from *Lentinus crinitus*, β-glucan from *Lentinus edodes*, extracts from* Lignosus rhinocerotis*, ribonuclease from *Lyophyllum shimeji*, chromatographic fractions from* Marasmius oreades*, hispolon from *Phellinus linteus*, antioxidant protein from *Pholiota nameko*, extracts from *Pleurotus ostreatus*, compounds from *Podostroma cornu-damae*, β-glucan from Poria cocos, polysaccharides from *Schizophyllum commune* 176. Yet the immunomodulation effects of fungi develop a novel insight for oncologists to generate better therapeutic avenues for cancer treatments 177. For example, a polysaccharide derived from a mushroom, *Boletus edulis*, increases the cytotoxic activity of the splenic natural killer cells and cytotoxic T lymphocytes, thus activating immune responses that inhibit the proliferation and growth of renal cancer in mice 177. Likewise, oral administration of β-1,3-Glucan derived from yeast (*Saccharomyces cerevisae*) in tumor-bearing mice stimulates granulocyte-macrophage progenitors and active cytokines such as IFN-γ, IL-1α, and IL-6, suppressing tumor progression 178.

**Figure 11:**
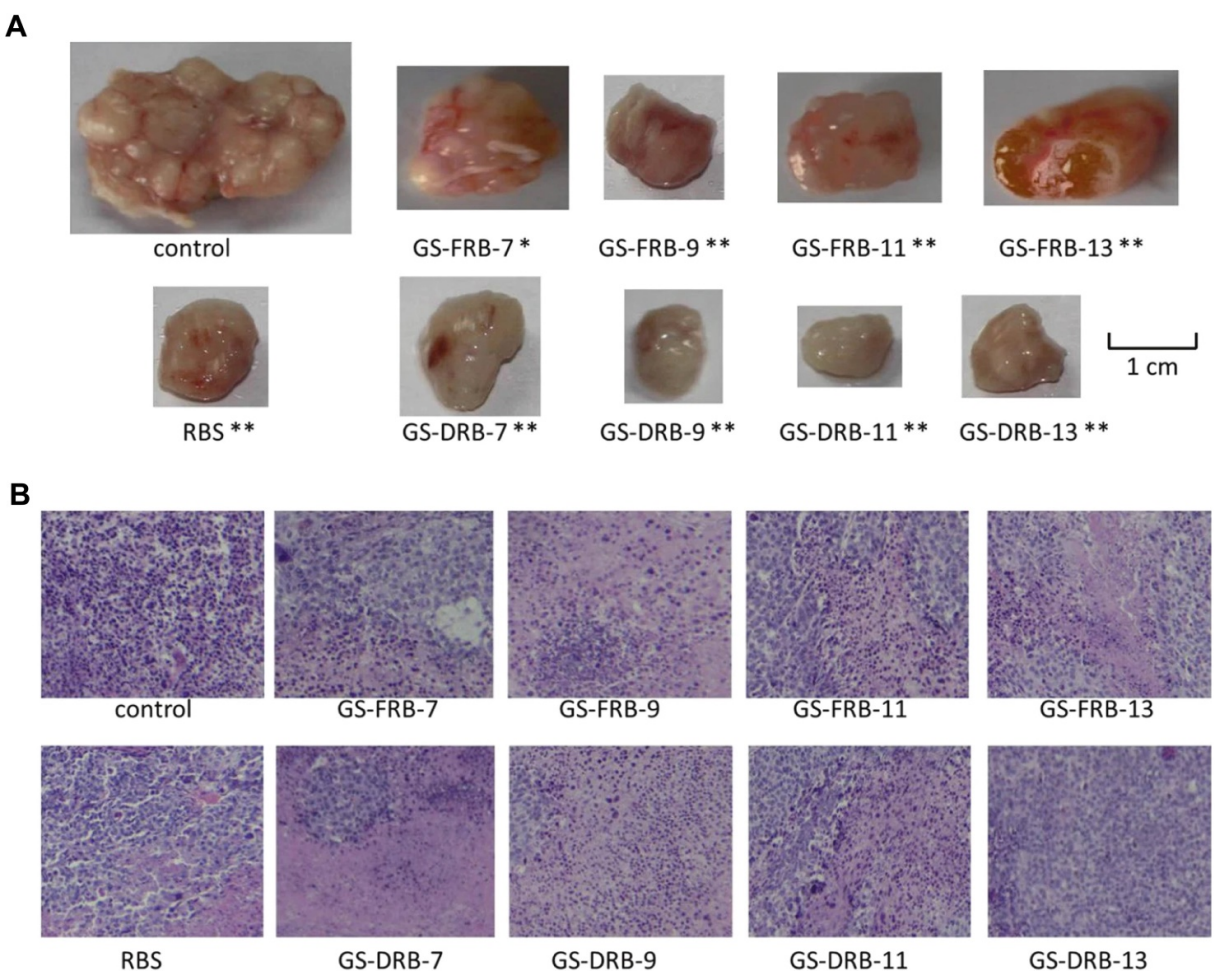
Polysaccharides from Ganoderma sinense suppress lung cancer in mice model. (A) Tumor volume. (B) H&E staining. Adapted with permission from 35, Copyright 2021, BioMed Central Ltd. Part of Springer Nature, CC BY 4.0.

In addition, fungal β-glucans accompanied with radiotherapy/chemotherapy have achieved positive therapeutic effects without obvious side effects on clinical trials of treating breast cancer, cervical cancer, gastrointestinal cancer, and prostate cancer 179. Polysaccharides from *Ganoderma sinense* modulate the activities of immune cells and secretion of cytokines 25, therefore suppressing H1299 non-small-cell lung cancer *ex vivo* and* in vivo* **(Figure 11)** 35. Similarly, D-Fraction from *Grifola frondosa* suppresses breast cancer both *in vivo* and* ex vivo*, as well as restricts lung metastases of breast cancer by modulating immune effects 180. Mangrove-derived endophytic fungi inhibit *in vitro* angiogenesis of lung cancer induced by HPV-16 E7 oncoprotein 181. Extracts from *Fomitopsis officinalis* not only exert apoptotic effects on cancer cells but also decrease tumor size and elongate the lifespan of tumor-bearing mice 182. Nevertheless, more discoveries are needed to explore the potential of fungi in cancer diagnosis and therapy.

### 4.5. Protozoa

**Figure 12:**
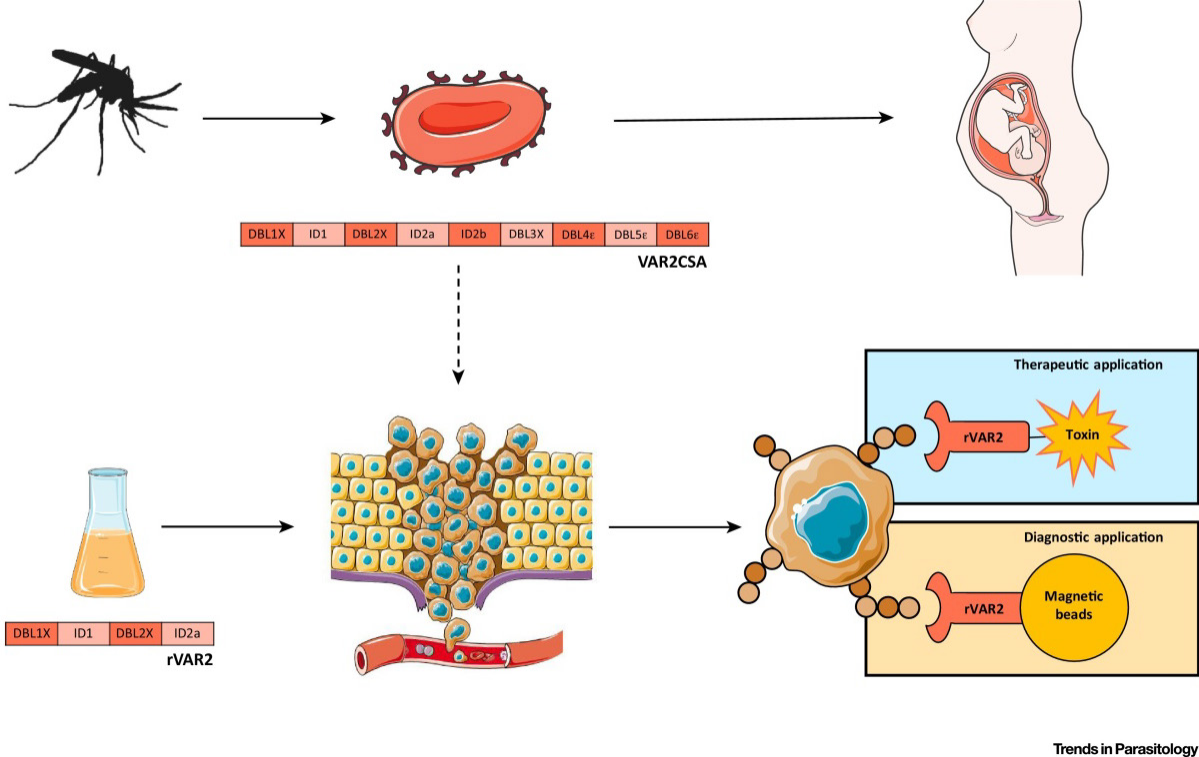
The *plasmodium* expressing VAR2CSA and recombinant VAR2CSA (rVAR2) can be applied to cancer diagnosis and therapy. Adapted with permission from 71, Copyright 2018, Elsevier Ltd.

**Figure 13:**
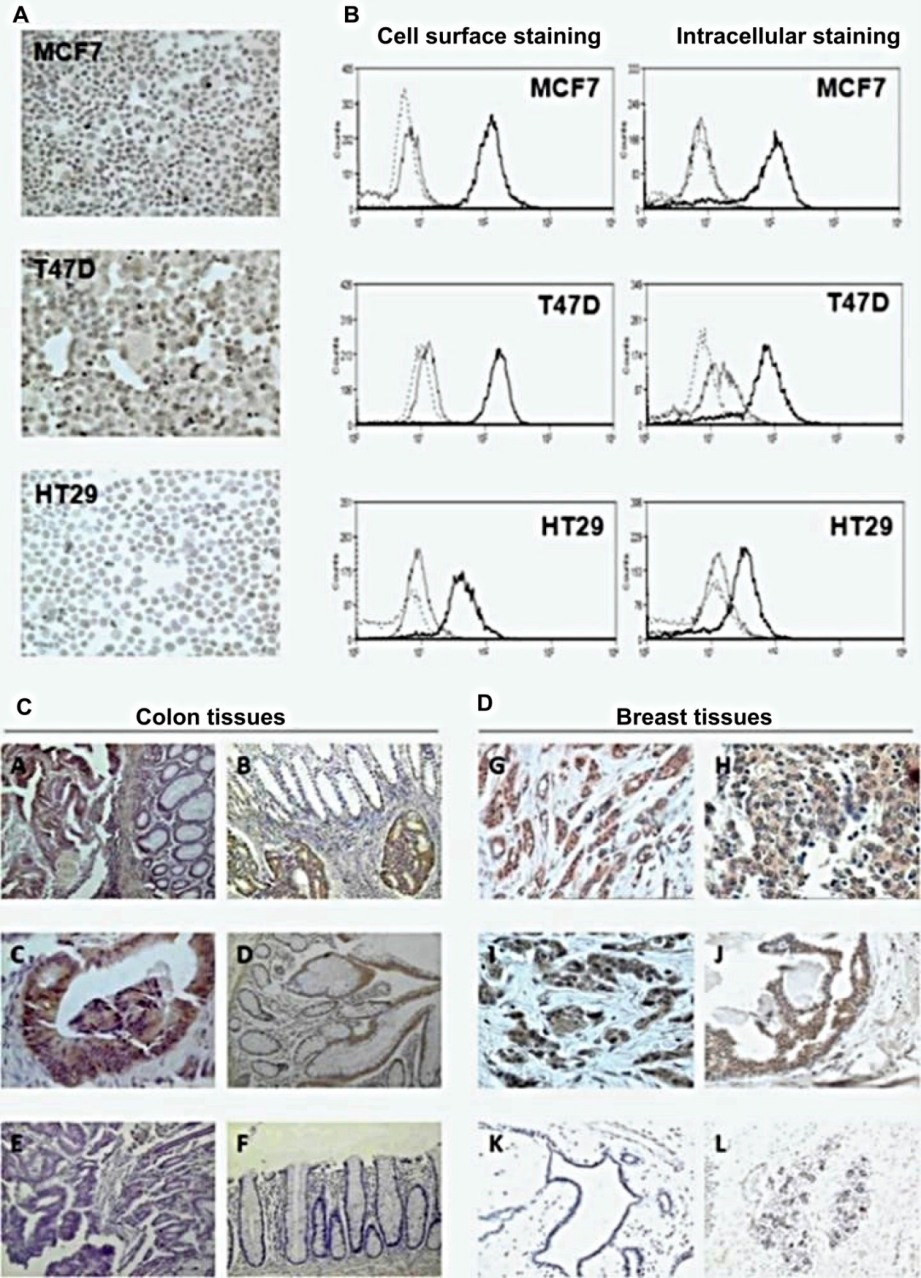
*Trypanosoma cruzi* extracts elicit protective immune response against chemically induced colon and mammary cancers. Adapted with permission from 36, Copyright 2015, UICC, John Wiley and Sons.

Due to the finding of the negative regulation impacts of protozoa on cancer progression 183, the anticancer action of protozoa and their products have been explored. Protozoa gradually gain their popular reputation not only in cancer treatment but also in cancer diagnosis and prognosis **(Table 8)**. For example, VAR2CSA expressed by *Plasmodium falciparum* is a binding protein to oncofetal chondroitin sulfate, which is widely expressed in many types of tumors. Thus, *Plasmodium falciparum* expressing VAR2CSA and recombinant VAR2CSA (rVAR2) can be used as a targeting probe, together with therapeutic molecules for cancers theranostics** (Figure 12)** 71. Leukocytes infected by *Theileria annulate* and *Theileria parva* potentially express cancer hallmarks including hypoxia inducible factor-1 alpha (HIF1α), transforming growth factor-beta (TGF-β), telomerase reverse transcriptase (TERT), murine double minute 2 (MDM2), nuclear factor-k-gene binding (NF-kB), *T. annulata* prolyl isomerase I gene (TaPIN1), matrix metalloproteinase-9 (MMP-9), tumor necrosis factor-alpha (TNF-α) and inhibitor of apoptosis protein (IAP), which are potential chemotherapeutic targets for cancer therapy 74. Likewise, *Toxoplasma gondii* and* Toxoplasma gondii*-derived molecules stimulate or block multiple signaling pathways such as TNF-α, NF-kB activity in modulating tumor microenvironment 112. Epimastigotes of *Trypanosoma cruzi* as vaccination could systematically activate macrophages, dendritic cells, CD4+ and CD8+ T cells, thereby increasing the NADPH oxidase activity to inhibit carcinogenesis **(Figure 13)** 36. These actions thus potentially inhibit cancers.

## 5. Potential mechanisms of microorganisms in cancer theranostics

### 5.1 The roles of microorganisms in tumor cells

**Figure 14:**
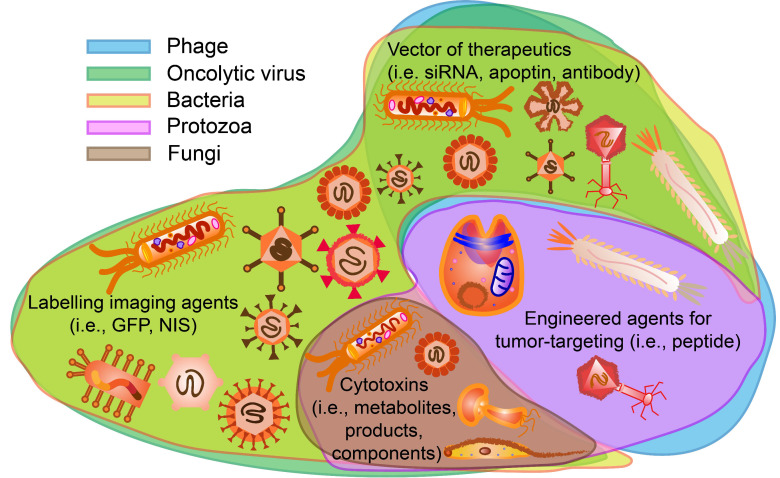
Potential functions of microorganisms on tumor cells for cancer theranostics. Phages and protozoa can display tumor-targeting agents such as peptides. Oncolytic viruses and bacteria can be labelled with imaging agents such as GFP, NIS and so on. Phages oncolytic viruses and bacteria are possible vectors for delivering certain therapeutics including apoptin, siRNA and antibodies. Oncolytic viruses, bacteria, fungi and protozoa contain or express cytotoxic components that can assist cancer therapy.

The roles of microorganisms in cancer theranostics can be divided into two major types: to regulate tumor cells and mediate immune cells. As shown in **Figure 14** and** Table 4-8**, the microorganisms above, including phages, oncolytic viruses, bacteria, fungi, and protozoa, present direct roles on tumor cells. Phages not only display tumor-targeting molecules (i.e., peptides, fragments) 116, 117, but also serve as drug delivery systems for cargos such as siRNA and antibodies 123, 124. Oncolytic viruses and bacteria play multi-functional roles in cancerous cells, including labeling them with imaging molecules (i.e., GFP, ^18^F-FDS and NIS) 51, 57, expressing cytotoxic components 50, 148, and delivering therapeutic agents 52, 153. Fungi only present cytotoxicity because their structure contains multiple polysaccharides 176, but protozoa only interact directly with tumor cells by expressing tumor-targeting proteins 71. In summary, the tumor-targeting effects of microorganisms are based on certain receptors on tumor cells 58, 71, 119, and the cytotoxic effects can be attributed to the stimulation of apoptotic (caspase 3/7, Bcl2, MAPK etc.) and autophagic pathways in tumor cells 53, 155, 184.

### 5.2 The roles of microorganisms in immune cells

The theranostic effects of microorganisms on cancer often rely on the cytokine networks or signaling pathways produced by immune cells in the host. Macrophages, dendritic cells, T cells, and NK cells are the most common types of immune cells triggered by microorganisms. Bacteria can stimulate the anti-tumoral effects through the use of macrophages to activate IL-1β/TNF-α signaling 148, and the use of fungi/fungi extract to activate IL-1α/IL-6/IFN-γ signaling 178. Dendritic cells can also excrete IL-1β to further enhance CD8+ T cells and NK cells to produce IFN-γ when phages 97, bacteria 148, or protozoa 36, 185 are used to treat diseases. The downstream signaling pathways of NFκB, STAT, and TLR triggered by CD8+ T cells, Treg cells, and NK cells also participate in the microorganism-based cancer therapy 34, 145, 186.

## 6. Clinical trials of microorganisms in cancer theranostics

**Figure 15:**
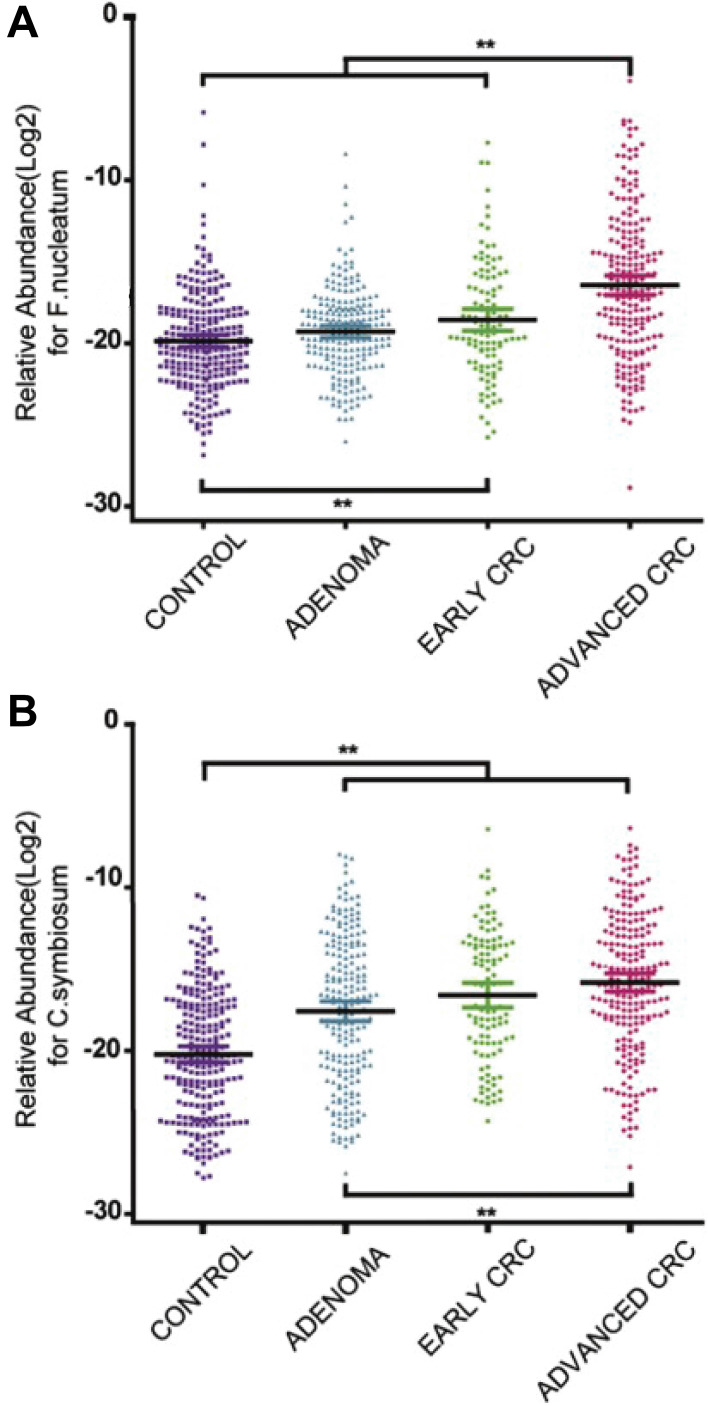
Abundance of *F. nucleatum* and *C. symbiosum* relative to colorectal cancer. Adapted with permission from 187. Copyright 2017, Elsevier B.V, CC BY-NC-ND 4.0.

**Figure 16:**
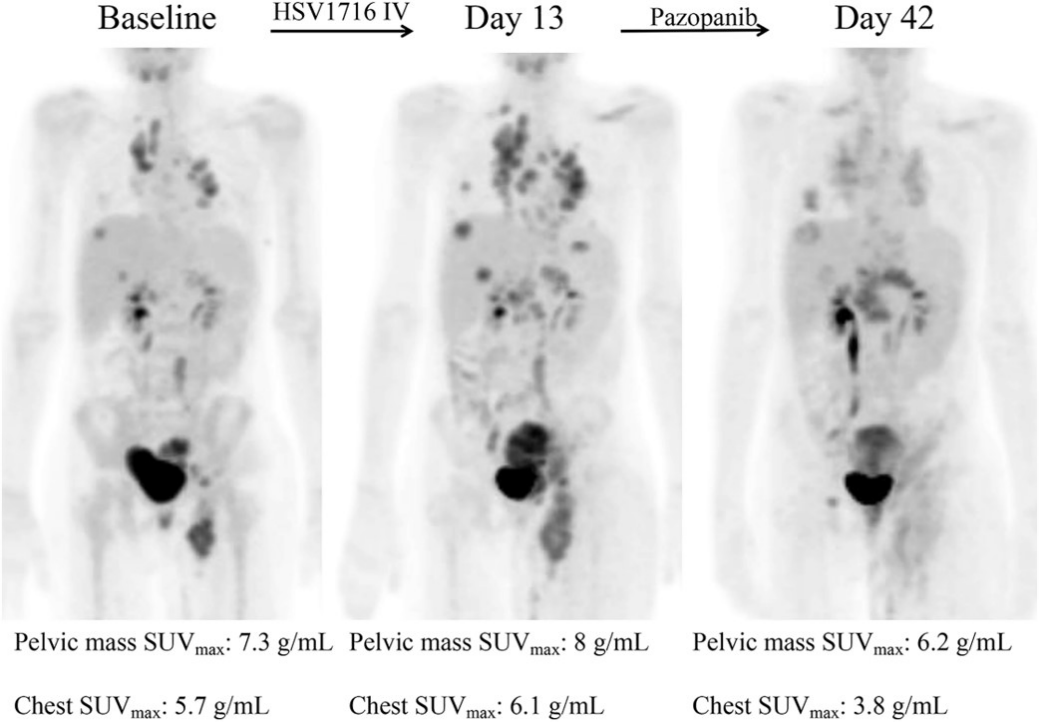
Decreased tumor metabolic activity shown in a patient after HSV1716 administration. Adapted with permission from 188. Copyright 2019, Elsevier Ltd, CC BY-NC-ND 4.0.

In addition, many clinical trials have been launched, ongoing, or completed in the field of microorganisms applied for cancer theranostics. We searched these trials registered in ClinicalTrials.gov (https://clinicaltrials.gov/) and EudraCT (https://www.clinicaltrialsregister.eu) and only listed all the completed studies that used microorganisms or engineered microorganisms directly in **Table 9**. The studies that used extracts and derived products of microorganisms are excluded from this table. As one can see, oncolytic viruses are mostly used for cancer therapy, especially in solid tumors, including ovarian cancer, bladder cancer, brain cancer, lung cancer, and gastrointestinal cancers. Moreover, to some extent, the anti-tumor effects of oncolytic viruses are somehow limited, and thus, they are employed with chemotherapeutic drugs together. Bacteria are mostly applied in cancer diagnosis or detecting the relationship between bacteria and cancers. Fang group screened the gut microbiota of colorectal cancer patients and found that the *Fusobacterium Nucleatum* and *Clostridium symbiosum* could be used to diagnose colorectal cancer (**Figure 15**, Table 9, Clinical Trial No. NCT02845973) 187. Fecal microbiota is also positively correlated with breast cancer and thus could be employed for early diagnosis of breast cancer (Table 9, Clinical Trial No. NCT01461070). Topical bacteriophage T4 endonuclease V shows positive effects in preventing the recurrence of skin cancer in patients undergoing kidney transplants (Table 9, Clinical Trial No. NCT00089180). Intravenous injection of oncolytic virus HSV-1 (HSV1716) is applied to chemotherapy for the treatment of different solid tumors, including cholangiocarcinoma, pancreatic neuroendocrine tumor, Ewing sarcoma, osteosarcoma, etc (**Figure 16**, Table 9, Clinical Trial No. NCT00931931) 188. Engineered *Listeria* are used for immunotherapy to treat of prostate cancer (Table 9, Clinical Trial No. NCT02625857). Protobics and low-bacteria diet act as adjuvants for potentially treating cancers. There are no completed clinical trials in these databases showing the application of fungi and protozoa in cancer theranostics. However, some products related to them are generated in this area. For instance, Imprime PGG, isolated from the cell wall of *Saccharomyces cerevisiae*, together with pembrolizumab, is being tested for its therapeutic effects on triple-negative breast cancer and melanoma (Clinical Trial No. NCT02981303).

## 7. Challenges and future perspectives

Traditional detection for microbiota is normally based on culturing specific microorganisms. According to the theory of microorganism detection, we should get a general knowledge of what the microorganisms need for survival; they can be cultured for us to observe their characteristic growth features. For instance, as for bacteria, we can only know their category on the premise of knowing their colony-forming units, such as filamentous colony, undulate colony, spore colony and etc. Therefore, we cannot identify the microorganisms if we do not have any records of their characteristics unless we do not have the technologies to culture them *in vitro*. To fill this gap, biologists invented a new measurement to identify the microorganisms on the basis of different metabolites of different microorganisms. This technology, called metabolome, can detect the profile of the metabolites in cells 189. Yet, it is still challenging for biologists to deeply get insights into microorganism verification. The most straitened circumstance is that microorganisms excrete similar (or even the same) metabolites so that we cannot distinguish them. Therefore, to further verify these microorganisms, a more specific and precise method is needed.

In recent years, the measurement of microbiota based on 16S sequencing has been adopted for microbiologists to explore the spectra of microorganisms and further classify them. However, 16S sequencing is contrived in light of the high conservative structure and function of the 16S rDNA in bacteria. The main drawbacks of 16S sequencing for detecting microbiota lie in the errors and low sensitivity in detecting heterogeneity of intra‐species 190. Also, it is not appropriate for other microorganisms such as viruses and fungi. To better solve these problems, whole-metagenome sequencing was launched to map the genomic regions precisely. Whole-metagenome sequencing gives new insights to deeply observe the genome in the microbiota as a result of the development of next-generation sequencing technologies. Global archives have been established and have stored millions of datasets for bacterial and viral whole-metagenome sequencing 191. There is no doubt that more categories will be identified and classified based on whole-metagenome sequencing in the future.

As massive microbiota spectra have been established, there comes the development of TME research and cancer therapy. On the one hand, we can obtain certain parts of tumor tissues and submit them to whole-metagenome sequencing to find out whether there is microbiota living in them or not. If so, we can substantially excogitate their roles in TME and cancer development, for example, to understand whether they help contribute to tumor proliferation, metastasis or inhibit them through secreting specific small molecules or cooperating with immune cells. On the other hand, we can compare the similarities and differences of different cancer TME and further establish datasets for TME microbiota. Doing so will help better understand the TME and build a foundation for cancer immunotherapy.

Even though many clinical trials have been down in the application of microorganisms for cancer theranostics, there are still some limitations and challenges. As we can see in **Table 9**, most completed trials directly using microorganisms in this field are using oncolytic viruses and bacteria. More explorations of phage, fungi, and protozoa in the clinical application should be investigated. Efforts should be made to evaluate not only the extracts and products of them but also the microorganisms themselves. Moreover, clinical tumor imaging and probe systems based on microorganisms are also limited and need further exploration. The most challenging part is the safety problems. Whether the microorganisms will only influence the tumor or not is important. If there will be side effects, how to decrease these effects after tumor treatment should be examined. Thus, more animal studies are welcome to discover the safety of microorganisms in individual bodies, in particular, to understand the immune response, the interaction between the introduced microorganisms and healthy tissues.

## 8. Conclusion

The applications of microorganisms for cancer theranostics have excited the oncologists in understanding the pathogenicity, diagnosis, progression, and treatment of cancer. Naturally, microorganisms reside in tumors, some of which are oncogenic, anti-tumoral, or just commensally residents. Many completed clinical trials have shown the diagnostic effects of microorganisms on the tumor. In addition, the anti-tumor functions of oncolytic viruses and bacteria have been widely launched clinically. However, more investigations should be done to evaluate the clinical values of phage, fungi, and protozoa. Due to the development of whole-metagenome sequencing, screening and identifying the specific microorganisms in certain tumor tissue has never been made easy like today. In this way, we can investigate the microbiota spectra of the tumor tissues and further distinguish their effects on cancer theranostics. Therefore, by uncovering the different impacts of the different microorganisms, we could deeply generate a precise probe, monitor, vaccine, or drug for cancer diagnosis and therapy.

## Figures and Tables

**Table 1:** Phages in TME and their potential functions

Phage	Tumor	Function in TME	Ref
Acinetobacter phage Acj61	Colorectal cancer	Unknown	96
Aeromonas phage PX29	Colorectal cancer	Unknown	96
Bacillus phage PfEFR‐5	Colorectal cancer	Host-*Bacillus cereus*	96
Clostridium phage phiCT9441A	Colorectal cancer	Unknown	96
Enterobacteria phage HK629	Liver metastasis of colorectal cancer	Unknown	96
Enterobacteria phage HK97	Liver metastasis of colorectal cancer	Unknown	96
Enterobacteria phage M13	Liver metastasis of colorectal cancer	Unknown	96
Enterobacteria phage mEp460	Liver metastasis of colorectal cancer	Unknown	96
Enterobacteria phage P1	Liver metastasis of colorectal cancer	Unknown	96
Enterobacteria phage P2	Colorectal cancer	Host-*Escherichia coli*	96
Enterobacteria phage P88	Colorectal cancer	Unknown	96
Enterobacteria phage VT2φ_272	Liver metastasis of colorectal cancer	Unknown	96
Enterobacteria phage λ	Colorectal cancer and liver metastasis	Host-*Escherichia coli*	96
Enterobacteria phage φ80	Liver metastasis of colorectal cancer	Host-*Escherichia coli*	96
Escherichia phage PBECO 4	Colorectal cancer	Unknown	96
Escherichia phage pro483	Liver metastasis of colorectal cancer	Unknown	96
Escherichia phage TL-2011b	Colorectal cancer and liver metastasis	Unknown	96
Lactobacillus phage Lb338-1	Colorectal cancer	Unknown	96
Mycobacterium phage Myrna	Colorectal cancer	Unknown	96
Phage cdtI DNA	Colorectal cancer and liver metastasis	Unknown	96
Prochlorococcus phage P-SSP7	Colorectal cancer	Unknown	96
Proteus phage vB_PmiM_Pm5461	Colorectal cancer	Host-*Proteus mirabilis*	96
Shigella phage SfII	Liver metastasis of colorectal cancer	Unknown	96
Shigella phage SfIV	Liver metastasis of colorectal cancer	Unknown	96
Staphylococcus phage StB20-like	Colorectal cancer	Unknown	96
Streptococcus phage A25	Colorectal cancer	Unknown	96
Streptococcus phage PH15	Colorectal cancer	Unknown	96
Streptococcus phage phiARI0462	Colorectal cancer	Host-*Streptococcus pneumoniae*	96
Streptococcus phage phiARI0923	Colorectal cancer	Host-*Streptococcus pneumoniae*	96
Streptococcus phage phiNJ2	Colorectal cancer	Unknown	96
Synechococcus phage S-SM2	Colorectal cancer and liver metastasis	Unknown	96
Uncultured crAssphage	Colorectal cancer	Unknown	96

**Table 2:** Other viruses in TME and their potential functions and therapeutics

Other Viruses	Tumor	Function	Therapeutic approach	Ref
Acanthamoeba polyphaga mouvirus	Colorectal cancer	Unknown		96
AcMNPV	Colorectal cancer and liver metastasis	Unknown		96
Cafeteria roenbergensis virus	Colorectal cancer and liver metastasis	Unknown		96
CMV	Colorectal cancer	Unknown		96
EBV	Burkitt's lymphoma, nasopharyngeal cancer, Hodgkin and non-Hodgkin's lymphoma	Oncogenic	Vaccine; Acyclovir	102
EBV	Colorectal cancer and liver metastasis	Unknown		96
Encephalomyocarditis virus	Liver metastasis of colorectal cancer	Unknown		96
HBV	Hepatocellular carcinoma	Oncogenic	Vaccine; Interferon; Antiviral agents	102
HCV	Hepatocellular carcinoma	Oncogenic	Vaccine; Interferon; Antiviral agents	102
HCV genotype 1	Liver metastasis of colorectal cancer	Unknown		96
HERV-K113	Colorectal cancer and liver metastasis	Unknown		96
HHV-6B	Colorectal cancer and liver metastasis	Unknown		96
HHV-7	Colorectal cancer and liver metastasis	Unknown		96
HPV	Cervical cancer, vaginal cancer, anal cancer	Oncogenic	Vaccine	102
HTLV-1	Adult T cell lymphoma	Oncogenic	No effective vaccine	102
KSHV	Kaposi's sarcoma, primary effusion lymphoma	Oncogenic	Antiviral agents	102
Lymphocystis disease virus	Colorectal cancer	Unknown		96
Megavirus chiliensis	Colorectal cancer	Unknown		96
MCV	Merkel cell carcinoma	Oncogenic	Unknown	102
Pandoravirus dulcis	Colorectal cancer and liver metastasis	Unknown		96
Pandoravirus neocaledonia	Colorectal cancer and liver metastasis	Unknown		96
Pandoravirus salinus	Colorectal cancer and liver metastasis	Unknown		96
Qinghai Himalayan marmot astrovirus	Colorectal cancer and liver metastasis	Unknown		96
Simian virus 40	Colorectal cancer	Unknown		96
Tipula oleracea nudivirus	Colorectal cancer and liver metastasis	Unknown		96
Torque teno midi virus 5	Liver metastasis of colorectal cancer	Unknown		96
Torque teno midi virus 9	Liver metastasis of colorectal cancer	Unknown		96
Torque teno virus 16	Colorectal cancer	Unknown		96
Torque teno virus 24	Colorectal cancer	Unknown		96

**Table 3:** Bacteria/Fungi/Protozoa in TME and their potential functions and therapeutics

Bacteria/Fungi/Protozoa		Tumor	Function	Therapeutic approach	Ref
Bacteria	*Anaerococcus mediterraneensis*	Colorectal cancer	Unknown		96
	*Bacillus cereus*	Colorectal cancer	Unknown		96
	*Bacteroides fragilis*	Colorectal cancer	Unknown		96
	*Enterococcus faecalis*	Colorectal cancer	Unknown		96
	*Escherichia coli*	Colorectal cancer and liver metastasis	Unknown		96
	*Fusobacterium hwasookii*	Colorectal cancer	Unknown		96
	*Fusobacterium nucleatum*	Colorectal cancer	Oncogenic	Antibiotics	107
	*Klebsiella pneumoniae*	Colorectal cancer and liver metastasis	Unknown		96
	*Porphyromonas gingivalis*	Colorectal cancer	Unknown		96
	*Prevotella denticola*	Colorectal cancer	Unknown		96
	*Streptococcus anginosus*	Colorectal cancer	Unknown		96
	*Streptococcus pneumoniae*	Colorectal cancer	Unknown		96
Fungi	*Ascomycota*	Pancreatic ductal adenocarcinoma	Unknown		111
	*Basidiomycota*	Pancreatic ductal adenocarcinoma	Unknown		111
	*Candida albicans*	Renal cell carcinoma; squamous cell carcinom	Oncogenic		109
	*Malassezia globosa*	Pancreatic ductal adenocarcinoma	Oncogenic		111
Protozoa	*Toxoplasma gondii*	Brain, lung, prostate, cervix, and endometrial cancers	Oncogenic	Anti-Toxoplasma drugs	112

**Table 4:** Phages applied for cancer diagnosis and therapy.

Phages	Technology/Mechanism	Diagnosis/Monitor	Therapy
M13 (e.g. M13mp19 116, 119, M13KO7 30, 117, 124, 192, etc.; T4 32; T7 46; fd phage 122; fd-tet 123	Page display	Screen Oligopeptides 116, antigen binding fragment 117 and gene-specific affibody 192 for cancer targeting imaging and diagnosis	Screen peptides for targeted therapy of cancer 119; Generating gene-targeting agents for cancer therapy 30; Generating monoclonal antibody for cancer chemotherapy 124; Generating vaccines;^32^, ^46^ Guiding the delivery of small interfering RNA (siRNA) for cancer gene therapy;^123^ Acting as immunomodulators 193 *in vivo*

**Table 5:** Oncolytic viruses applied for cancer diagnosis and therapy.

Oncolytic virus	Technology/Mechanism	Diagnosis/ Therapy
Adenovirus 133-135; HSV-1 136; Measles virus 137; Newcastle disease virus 53; Parvovirus 138; Vaccinia virus 139; VSV 140-142	Adding luciferase genes, fluorescent proteins or radio-labelled substrates into virus	Bioluminescence imaging, fluorescence imaging and nuclear medicine-based imaging 51
Adenovirus 52; HSV-1 136; Measles virus 137; Newcastle disease virus 53; Poliovirus 138; Vaccinia virus 145; VSV 140-142; Reovirus 194	Anti-proliferation, anti-apoptosis and immune modulators; Vectors for gene therapy	Acting as an oncolytic agent 50, 145; Applied for cancer immunotherapy 144; Delivery of molecules, siRNA and shRNA for cancer gene therapy 52, 146

**Table 6:** Bacteria applied for cancer diagnosis and therapy.

Bacteria	Technology/Mechanism	Diagnosis/Therapy
Escherichia coli 57	^18^F-FDS uptake by bacteria strain	Nuclear medicine-based imaging
Escherichia coli 149	Stimulating apoptotic and autophagic effects by products	Anti-tumor effects on cell lines *in vitro*
Listeria monocytogenes;^34^, ^152^ Salmonella Typhimurium 150	Stimulating apoptotic and autophagic effects by stains and products; Modulating immune cells and cytokine/chemokine networks	Acting as an oncolytic agent; Applied for cancer immunotherapy *in vivo*
Clostridium sp. 153; Escherichia coli 153; Listeria monocytogenes 152; Pseudomonas 154; Salmonella Typhimurium 151; Salmonella sp. 153	Vectors for gene therapy	Delivery of tumor antigen, DNA plasmids, siRNA and shRNA for cancer gene therapy *in vivo*

**Table 7:** Fungi applied for cancer therapy.

Fungi	Mechanism	Therapy
Agaricus bisporus 176; Agaricus blazei 176; Amauroderma rude 176; Amauroderma rugosum 172; Antrodia camphorate 173; Clitocybe alexandri 169; Coprinus comatus 174; Cordyceps militaris 158; Coriolus versicolor 156; Daldinia concentrica 163; Flammulina velutipes 175; Fomes fomentarius 176; Fomitopsis officinalis 182; Fuscoporia torulosa 176; Ganoderma lucidum 157; Ganoderma sinense 35; Grifola frondose 180; Hericium erinaceum 165; Hypsizigus marmoreus 176; Inonotus obliquus 161; Laetiporus sulphureus 159; Lentinus crinitus 176; Lentinus edodes 176; Lepista inversa 169; Lignosus rhinocerotis 176; Lyophyllum shimeji 176; Marasmius oreades 176; Paecilomyces japonica 162; Phellinus linteus 176; Pholiota nameko 176; Pleurotus eryngii 160; Pleurotus ostreatus 176; Podostroma cornu-damae 176; Poria cocos 176; Russula delica 166; Russula lepida 167; Schizophyllum commune 176; Tricholoma mongolicum 168; Xylaria psidii 170; Xylaria schweinitzii 164; Xylaria sp.^171^;	Stimulating apoptotic and autophagic effects by extracts	Anti-tumoral effects on cell lines *in vitro*
Antrodia camphorate 176; Auricularia auricularia-judae 195; Coriolus versicolor 196; Fomitopsis officinalis 182; Ganoderma sinense 35; Grifola frondose 180; Lentinus edodes 176; Pleurotus ostreatus 197; Schizophyllum commune 176	Modulating immune cells and cytokine/chemokine networks 176	Decreasing tumor size, inhibiting metastasis and elongating lifespan of tumor bearing animals (Acting as an oncolytic agent; Therapeutics for cancer immunotherapy *in vivo*)

**Table 8:** Protozoa applied for cancer diagnosis and therapy.

Protozoa	Technology/Mechanism	Diagnosis/Monitor	Therapy
Plasmodium falciparum 71; Theileria annulate 74; Theileria parva 74; Toxoplasma gondii 185, 186	Targeting tumor protein	Detecting tumor	Drugs for tumor targeting therapy
Toxoplasma gondii 198; Trypanosoma cruzi 36	Extracts or components modulate immune cells and cytokine/chemokine networks		Inhibiting tumor growth; Generating immune vaccines

**Table 9:** Clinical Trials of microorganisms applied for cancer theranostics

Microorganisms	Tumor type	Clinical Studies	Year	Database (ID)
T4 phage	Skin Cancer	T4N5 Liposomal Lotion in Preventing the Recurrence of Nonmelanoma Skin Cancer in Patients Who Have Undergone a Kidney Transplant	2004-2007	ClinicalTrials.gov [a] (NCT00089180)
MV-CEA, and MV-NIS (Oncolytic virus)	Ovarian cancer	Recombinant Measles Virus Vaccine Therapy and Oncolytic Virus Therapy in Treating Patients with Progressive, Recurrent, or Refractory Ovarian Epithelial Cancer or Primary Peritoneal Cancer	2004-2017	ClinicalTrials.gov (NCT00408590)
GL-ONC1 (Oncolytic virus)	Solid Tumors	Safety Study of GL-ONC1, an Oncolytic Virus, in Patients with Advanced Solid Tumors	2008-2015	ClinicalTrials.gov (NCT00794131)
CG0070 (Oncolytic virus)	Bladder Cancer	Safety and Efficacy of CG0070 Oncolytic Virus Regimen for High Grade NMIBC After BCG Failure	2015-2019	ClinicalTrials.gov (NCT02365818)
TBI-1401 (HF10) (Oncolytic virus)	Solid Tumor	A Study of TBI-1401(HF10) in Patients with Solid Tumors with Superficial Lesions	2015-2107	ClinicalTrials.gov (NCT02428036)
Enadenotucirev (Oncolytic virus)	Ovarian Cancer	Phase I / Dose Expansion Study of Enadenotucirev in Ovarian Cancer Patients	2014-2019	ClinicalTrials.gov (NCT02028117)
Enadenotucirev (Oncolytic virus)	Solid Tumours	Phase I / II Study of Enadenotucirev by Sub-acute Fractionated IV Dosing in Cancer Patients	2012-2016	ClinicalTrials.gov (NCT02028442)
G207 (Oncolytic virus)	Brain Cancer	Safety and Effectiveness Study of G207, a Tumor-Killing Virus, in Patients with Recurrent Brain Cancer	2001-2003	ClinicalTrials.gov (NCT00028158)
Colo-Ad1 (Oncolytic virus)	Colon Cancer; Non-small Cell Lung Cancer; Bladder Cancer; Renal Cell Carcinoma	Mechanism of Action Trial of ColoAd1	2013-2016	ClinicalTrials.gov (NCT02053220)
ONCOS-102 (Oncolytic virus)	Solid Tumour	ONCOS-102 (Previously CGTG-102) for Therapy of Advanced Cancers	2012-2013	ClinicalTrials.gov (NCT01598129)
HSV1716 (Oncolytic virus)	Solid Tumour	HSV1716 in Patients with Non-Central Nervous System (Non-CNS) Solid Tumors	2010-2018	ClinicalTrials.gov (NCT00931931)
DNX-2401 (Oncolytic virus)	Brain Tumors	DNX-2401 With Interferon Gamma (IFN-γ) for Recurrent Glioblastoma or Gliosarcoma Brain Tumors	2014-2018	ClinicalTrials.gov (NCT02197169)
VCN-01	Solid Tumors	Phase I Dose Escalation Study of Intravenous VCN-01 With or Without Gemcitabine and Abraxane® in Patients with Advanced Solid Tumors	2014-2020	ClinicalTrials.gov (NCT02045602)
Ad-MAGEA3, MG1-MAGEA3 (Oncolytic virus)	Non-Small Cell Lung Cancer	Oncolytic MG1-MAGEA3 With Ad-MAGEA3 Vaccine in Combination with Pembrolizumab for Non-Small Cell Lung Cancer Patients	2017-2020	ClinicalTrials.gov (NCT02879760)
HSV1716 (Oncolytic virus)	Mesothelioma	Intrapleural Administration of HSV1716 to Treat Patients with Malignant Pleural Mesothelioma	2012-2016	ClinicalTrials.gov (NCT01721018)
REOLYSIN® (Oncolytic virus)	Colorectal Cancer	Study of REOLYSIN® in Combination with FOLFIRI and Bevacizumab in FOLFIRI Naive Patients With KRAS Mutant Metastatic Colorectal Cancer	2010-2018	ClinicalTrials.gov (NCT01274624)
GL-ONC1 (Oncolytic virus)	Cancer of Head and Neck	Safety Study of Attenuated Vaccinia Virus (GL-ONC1) with Combination Therapy in Head & Neck Cancer	2012-2015	ClinicalTrials.gov (NCT01584284)
JX-594 (Oncolytic virus)	Hepatic Carcinoma	A Study of Recombinant Vaccinia Virus to Evaluate the Safety and Efficacy of a Transdermal Injection Within the Tumor of Patients with Primary or Metastatic Hepatic Carcinoma	2006-2007	ClinicalTrials.gov (NCT00629759)
JX-594 (Oncolytic virus)	Liver Cancer	A Phase 2b Study of Modified Vaccinia Virus to Treat Patients Advanced Liver Cancer Who Failed Sorafenib	2008-2011	ClinicalTrials.gov (NCT01387555)
DNX-2401 (Oncolytic virus)	Brain Cancer	Combination Adenovirus + Pembrolizumab to Trigger Immune Virus Effects	2016-2021	ClinicalTrials.gov (NCT02798406)
REOLYSIN® (Oncolytic virus)	Non-small Cell Lung Carcinoma	Phase 2 Study of REOLYSIN® in Combination with Paclitaxel and Carboplatin for Non-Small Cell Lung Cancer With KRAS or EGFR Activation	2009-2015	ClinicalTrials.gov (NCT00861627)
CVA21 (Oncolytic virus)	Uveal Melanoma; Liver Metastases	CAVATAK® and Ipilimumab in Uveal Melanoma Metastatic to the Liver (VLA-024 CLEVER)	2018-2019	ClinicalTrials.gov (NCT03408587)
JX-594 (Oncolytic virus)	Solid Tumors	Safety Study of Recombinant Vaccinia Virus to Treat Refractory Solid Tumors	2008-2014	ClinicalTrials.gov (NCT00625456)
VCN-01 (Oncolytic virus)	Pancreatic Adenocarcinoma	A Phase I Dose Escalation Study of Intratumoral VCN-01 Injections with Gemcitabine and Abraxane® in Patients with Advanced Pancreatic Cancer	2014-2018	ClinicalTrials.gov (NCT02045589)
JX-594 (Oncolytic virus)	Solid Tumors	Safety Study of Recombinant Vaccinia Virus to Treat Refractory Solid Tumors in Pediatric Patients	2010-2014	ClinicalTrials.gov (NCT01169584)
REOLYSIN® (Oncolytic virus)	Malignant Glioma	Safety and Efficacy Study of REOLYSIN® in the Treatment of Recurrent Malignant Gliomas	2006-2010	ClinicalTrials.gov (NCT00528684)
GL-ONC1 (Oncolytic virus)	Peritoneal Carcinomatosis	A Study of GL-ONC1, an Oncolytic Vaccinia Virus, in Patients with Advanced Peritoneal Carcinomatosis	2012-2014	ClinicalTrials.gov (NCT01443260)
TBI-1401 (HF10) (Oncolytic virus)	Melanoma	A Study of Combination With TBI-1401(HF10) and Ipilimumab in Japanese Patients with Unresectable or Metastatic Melanoma	2017-2018	ClinicalTrials.gov (NCT03153085)
HF10 (Oncolytic virus)	Melanoma	A Study of Combination Treatment with HF10 and Ipilimumab in Patients With Unresectable or Metastatic Melanoma	2014-2018	ClinicalTrials.gov (NCT02272855)
JX-594 (Oncolytic virus)	Colorectal Carcinoma	Recombinant Vaccinia Virus Administered Intravenously in Patients with Metastatic, Refractory Colorectal Carcinoma	2012-2015	ClinicalTrials.gov (NCT01394939)
REOLYSIN® (Oncolytic virus)	Sarcomas	Safety and Efficacy Study of REOLYSIN® in the Treatment of Bone and Soft Tissue Sarcomas Metastatic to the Lung	2007-2011	ClinicalTrials.gov (NCT00503295)
CVA21(Oncolytic virus)	Melanoma	A Study of Intratumoral CAVATAK™ in Patients With Stage IIIc and Stage IV Malignant Melanoma (VLA-007 CALM )	2011-2016	ClinicalTrials.gov (NCT01227551)
JX-594 (Oncolytic virus)	Melanoma	A Study of Recombinant Vaccinia Virus to Treat Malignant Melanoma	2007-2009	ClinicalTrials.gov (NCT00429312)
CVA21(Oncolytic virus)	Melanoma	A Safety Study of Two Intratumoural Doses of Coxsackievirus Type A21 in Melanoma Patients (PSX-X03)	2007-2009	ClinicalTrials.gov (NCT00438009)
ParvOryx (Oncolytic virus)	Glioblastoma	Parvovirus H-1 (ParvOryx) in Patients With Progressive Primary or Recurrent Glioblastoma Multiforme.	2011-2015	ClinicalTrials.gov (NCT01301430)
REOLYSIN® (Oncolytic virus)	Pancreatic Adenocarcinoma	Study of Pembrolizumab With REOLYSIN® and Chemotherapy in Patients With Advanced Pancreatic Adenocarcinoma	2015-2018	ClinicalTrials.gov (NCT02620423)
DNX2401 (Oncolytic virus)	Glioblastoma	Virus DNX2401 and Temozolomide in Recurrent Glioblastoma	2013-2017	ClinicalTrials.gov (NCT01956734)
REOLYSIN® (Oncolytic virus)	Ovarian Epithelial, Fallopian Tube, or Primary Peritoneal Cancer	Paclitaxel With or Without Viral Therapy in Treating Patients with Recurrent or Persistent Ovarian Epithelial, Fallopian Tube, or Primary Peritoneal Cancer	2010-2020	ClinicalTrials.gov (NCT01199263)
MV-NIS (Oncolytic virus)	Myeloma	UARK 2014-21 A Phase II Trial of Oncolytic Virotherapy by Systemic Administration of Edmonston Strain of Measles Virus	2015-2019	ClinicalTrials.gov (NCT02192775)
Pexa Vec (Oncolytic virus)	Hepatocellular Carcinoma	Hepatocellular Carcinoma Study Comparing Vaccinia Virus Based Immunotherapy Plus Sorafenib vs Sorafenib Alone	2015-2020	ClinicalTrials.gov (NCT02562755)
JX-594 (Oncolytic virus)	Hepatocellular Carcinoma	A Study of Recombinant Vaccinia Virus to Treat Unresectable Primary Hepatocellular Carcinoma	2008-2013	ClinicalTrials.gov (NCT00554372)
MV-NIS (Oncolytic virus)	Mesothelioma	Intrapleural Measles Virus Therapy in Patients with Malignant Pleural Mesothelioma	2011-2019	ClinicalTrials.gov (NCT01503177)
T-VEC (Oncolytic virus)	Melanoma	A Study of Talimogene Laherparepvec in Stage IIIc and Stage IV Malignant Melanoma	2005-2008	ClinicalTrials.gov (NCT00289016)
ONCOS-102	Melanoma	A Pilot Study of Sequential ONCOS-102, an Engineered Oncolytic Adenovirus Expressing GMCSF, and Pembrolizumab in Patients with Advanced or Unresectable Melanoma Progressing After Programmed Cell Death Protein 1 (PD1) Blockade	2016-2020	ClinicalTrials.gov (NCT03003676)
ParvOryx (Oncolytic virus)	Pancreatic Cancer	A non-controlled, single arm, open label, Phase II study of intravenous and intratumoral administration of ParvOryx in patients with metastatic, inoperable pancreatic cancer	2015-2018	EudraCT [b] (2015-001119-11)
ParvOryx (Oncolytic virus)	Glioblastoma	Phase I/IIa study of intratumoral/intracerebral or intravenous/intracerebral administration of PArvovirus H-1 (ParvOryx) in patients with progressive primary or recurrent glioblastoma multiforme	2011-2015	EudraCT (2011-000572-33)
HSV1716 (Oncolytic virus)	Pleural mesothelioma	A Phase I/IIa Study Of The Safety, Tolerability And Biological Effect Of Single And Repeat Administration Of The Selectively Replication-Competent Herpes Simplex Virus Hsv1716 Into The Tumour-Bearing Pleural Cavity (Intrapleural) In Patients With Inoperable Malignant Pleural Mesothelioma.	2012-2016	EudraCT (2010-024496-37)
Pexa-Vec (Oncolytic virus)	Hepatocellular carcinoma	A phase I/IIa trial to evaluate the safety and efficacy of the combination of the oncolytic immunotherapy Pexa-Vec with the PD-1 receptor blocking antibody nivolumab in the first-line treatment of advanced hepatocellular carcinoma (HCC)	2018-2020	EudraCT (2016-000085-32)
ONCOS-102 (Oncolytic virus)	Pleural mesothelioma	A randomised Phase II open-label study with a Phase Ib safety lead-in cohort of ONCOS-102, an immune-priming GM-CSF coding oncolytic adenovirus, and pemetrexed/cisplatin in patients with unresectable malignant pleural mesothelioma	2018-2019	EudraCT (2015-005143-13)
T-VEC (Oncolytic virus)	Melanoma	A Phase 1b/2, Multicenter, Open-label Trial to Evaluate the Safety and Efficacy of Talimogene Laherparepvec and Ipilimumab Compared to Ipilimumab Alone in Subjects With Unresected, Stage IIIB-IV Melanoma	2014-2021	EudraCT (2012-000307-32)
VNP20009 (Bacteria)	Cancer	Treatment of Patients With Cancer With Genetically Modified Salmonella Typhimurium Bacteria	2000-2002	ClinicalTrials.gov (NCT00004988)
Lactobacillus plantarum HEAL 19 (Bacteria)	Rectal Cancer	Action of Synbiotics on Irradiated GI Mucosa in Rectal Cancer Treatment	2008-2015	ClinicalTrials.gov (NCT03420443)
Intestine bacteria	Breast Cancer	Intestine Bacteria and Breast Cancer Risk	2011-2020	ClinicalTrials.gov (NCT01461070)
Gut bacteria	Breast Cancer	Engineering Gut Microbiome to Target Breast Cancer	2017-2020	ClinicalTrials.gov (NCT03358511)
Gut bacteria	Colorectal cancer	Study of Fecal Bacteria in Early Diagnosis of Colorectal Cancer	2012-2017	ClinicalTrials.gov (NCT02845973)
Bacteria vaccine	Cancer	A Phase 1 Study of Mixed Bacteria Vaccine (MBV) in Patients with Tumors Expressing NY-ESO-1 Antigen	2007-2013	ClinicalTrials.gov (NCT00623831)
C. novyi-NT (Bacteria)	Solid Tumor	Safety Study of Intratumoral Injection of Clostridium Novyi-NT Spores to Treat Patients With Solid Tumors That Have Not Responded to Standard Therapies	2013-2017	ClinicalTrials.gov (NCT01924689)
Colistimethate sodium (Bacteria)	Haematological Malignancies	A Study of DEcolonization in Patients with HAematological Malignancies (DEHAM)	2017-2017	ClinicalTrials.gov (NCT02966457)
Bacteria	Malignant Neoplasm	Peritoneal Bacterial Contamination Following Resection With Closed or Open Rectal Stump for Left-sided Cancer	2014-2014	ClinicalTrials.gov (NCT02527382)
Bacteria	Breast Cancer	Effects of Chemotherapy on Intestinal Bacteria in Patients With Newly Diagnosed Breast Cancer	2014-2018	ClinicalTrials.gov (NCT02370277)
AG013 (Bacteria)	Head and Neck Cancer	Study to Assess Safety and Tolerability of AG013 in Oral Mucositis in Subjects Receiving Induction Chemotherapy for the Treatment of Cancers of the Head and Neck	2009-2012	ClinicalTrials.gov (NCT00938080)
Oral bacteria	Pancreatic Cancer	Oral Microbiome and Pancreatic Cancer	1992-2010	ClinicalTrials.gov (NCT03302637)
La1, BB536 (Bacteria)	Colorectal Cancer	Probiotics In Colorectal Cancer Patients	2006-2007	ClinicalTrials.gov (NCT00936572)
Bl-04, NCFM (Bacteria)	Colon cancer	Using Probiotics to Reactivate Tumor Suppressor Genes in Colon Cancer	2010-2016	ClinicalTrials.gov (NCT03072641)
JNJ-64041809 (Bacteria)	Prostate Cancer	Safety & Immunogenicity of JNJ-64041809, a Live Attenuated Double-deleted Listeria Immunotherapy, in Participants With Metastatic Castration-resistant Prostate Cancer	2015-2018	ClinicalTrials.gov (NCT02625857)
Bacteria	Leukemia; Sarcoma; Neuroblastoma	The Effectiveness of the Neutropenic Diet in Pediatric Oncology Patients	2007-2017	ClinicalTrials.gov (NCT00726934)
Bacteria	Skin Cancer	Observational Study to Investigate Surgical Site Infection in Ulcerated Skin Cancers	2019-2020	ClinicalTrials.gov (NCT03782727)
Bacteria	Gastric Cancer	Gastric Cancer Precursor Lesions (GCPL) Study	2017-2020	ClinicalTrials.gov (NCT03188406)
Intestinal microbiome	Gastric Cancer	Intestinal Microbiome After Gastrectomy	2018-2019	ClinicalTrials.gov (NCT03418428)
Bacteria	Colorectal cancer, Stomach cancer, Pancreatic Cancer	Tracheal Colonization and Outcome After Major Abdominal Cancer Surgery	2008-2012	ClinicalTrials.gov (NCT04002128)
Bacteria	Colorectal cancer	Synbiotics and Gastrointestinal Function Related Quality of Life After Colectomy for Cancer	2010-2015	ClinicalTrials.gov (NCT01479907)
Bacteria	Colorectal Cancer	Microbiota-anastomotic Leak Among Colorectal Surgery Patients: Pilot Study	2018-2018	ClinicalTrials.gov (NCT03496441)
Fluoroquinolone Resistant Enteric Bacteria	Prostate cancer	Incidence of Fluoroquinolone Resistant Bacteria in Patients Undergoing Prostate Biopsy	2015-2016	ClinicalTrials.gov (NCT02140502)
Fecal Microbiota	Leukemia	PreventiOn of DYSbioSis Complications With Autologous FMT in AML Patients	2016-2018	ClinicalTrials.gov (NCT02928523)
Gut microbiome	Colorectal Adenoma	Ginger and Gut Microbiome	2018-2020	ClinicalTrials.gov (NCT03268655)
Probiotics (Bacteria)	Hepatocellular Carcinoma	Influence of Probiotics Administration Before Liver Resection in Liver Disease	2013-2018	ClinicalTrials.gov (NCT02021253)
BCG (Bacteria)	Bladder cancer	A Phase III Randomized, Open-Label, Multi-Center, Global Study of Durvalumab and Bacillus Calmette-Guerin (BCG) Administered as Combination Therapy Versus BCG Alone in High-Risk, BCG Naïve Non Muscle Invasive Bladder Cancer Patients	2017-2019	EudraCT (2017-002979-26)

^a)^ ClinicalTrials.gov (https://clinicaltrials.gov/); ^b)^ EudraCT (https://www.clinicaltrialsregister.eu)

## Abbreviations

Bl2: B cell lymphoma/leukemia 2
CLRs: C-type lectin receptors
EBV: Epstein-Barr virus
EBNA-1: EBV-determined nuclear anti gen 1
EBNA-2: EBV-determined nuclear anti gen 2
FDS: fluorodeoxysorbitol
GFP: green fluorescent protein
HIF1α: hypoxia inducible factor-1 alpha
HIV: human immunodeficiency virus
IAP: inhibitor of apoptosis protein
IONP: iron oxide nanoparticles
JAK: Janus kinases
LMP-1: latent membrane protein1
LMP-2: latent membrane protein 2
MAPK: mitogen-activated protein kinase
MDM2: murine double minute 2
MCV: Merkel cell polyomavirus
MMP-9: matrix metalloproteinase-9
NF-kB: nuclear factor-k-gene binding
NIS: sodium/iodide symporter
NK: natural killer
NLRs: nucleotide oligomerization domain (NOD)-like receptors
QD: quantum dots
RLRs: retinoic acid-inducible gene 1 (RIG-I)-like receptors
STAT: signal transducers and transcription activator
TaPIN1: *T. annulata* prolyl isomerase I gene
TERT: telomerase reverse transcriptase
TGF-β: transforming growth factor-beta
TLRs: Toll-like receptors
TME: tumor microenvironment
TNF-α: tumor necrosis factor-alpha

## References

[1] Bray F, Ferlay J, Soerjomataram I, Siegel RL, Torre LA, Jemal A (2018). Global cancer statistics 2018: GLOBOCAN estimates of incidence and mortality worldwide for 36 cancers in 185 countries. CA Cancer J Clin.

[2] Najafi M, Goradel NH, Farhood B, Salehi E, Solhjoo S, Toolee H (2019). Tumor microenvironment: Interactions and therapy. J Cell Physiol.

[3] Jobin C (2018). Precision medicine using microbiota. Science.

[4] El-Jurdi N, Ghannoum MA (2017). The mycobiome: Impact on health and disease states. Microbiol Spectr.

[5] Dzutsev A, Goldszmid RS, Viaud S, Zitvogel L, Trinchieri G (2015). The role of the microbiota in inflammation, carcinogenesis, and cancer therapy. Eur J Immunol.

[6] Breban M (2016). Gut microbiota and inflammatory joint diseases. Joint Bone Spine.

[7] Serrano-Villar S, Rojo D, Martinez-Martinez M, Deusch S, Vazquez-Castellanos JF, Sainz T (2016). HIV infection results in metabolic alterations in the gut microbiota different from those induced by other diseases. Sci Rep.

[8] Hsiao EY, McBride SW, Hsien S, Sharon G, Hyde ER, McCue T (2013). Microbiota modulate behavioral and physiological abnormalities associated with neurodevelopmental disorders. Cell.

[9] Qin N, Yang F, Li A, Prifti E, Chen Y, Shao L (2014). Alterations of the human gut microbiome in liver cirrhosis. Nature.

[10] Arthur JC, Jobin C (2011). The struggle within: microbial influences on colorectal cancer. Inflamm Bowel Dis.

[11] van der Velden WJ, Netea MG, de Haan AF, Huls GA, Donnelly JP, Blijlevens NM (2013). Role of the mycobiome in human acute graft-versus-host disease. Biol Blood Marrow Transplant.

[12] Qin J, Li Y, Cai Z, Li S, Zhu J, Zhang F (2012). A metagenome-wide association study of gut microbiota in type 2 diabetes. Nature.

[13] Dzutsev A, Badger JH, Perez-Chanona E, Roy S, Salcedo R, Smith CK (2017). Microbes and cancer. Annu Rev Immunol.

[14] Kahrstrom CT, Pariente N, Weiss U (2016). Intestinal microbiota in health and disease. Nature.

[15] Naik S, Bouladoux N, Linehan JL, Han SJ, Harrison OJ, Wilhelm C (2015). Commensal-dendritic-cell interaction specifies a unique protective skin immune signature. Nature.

[16] O'Brien S, Fothergill JL (2017). The role of multispecies social interactions in shaping Pseudomonas aeruginosa pathogenicity in the cystic fibrosis lung. FEMS Microbiol Lett.

[17] Arweiler NB, Netuschil L (2016). The oral microbiota. Adv Exp Med Biol.

[18] Di Pilato V, Freschi G, Ringressi MN, Pallecchi L, Rossolini GM, Bechi P (2016). The esophageal microbiota in health and disease. Ann N Y Acad Sci.

[19] Alarcon T, Llorca L, Perez-Perez G (2017). Impact of the microbiota and gastric disease development by Helicobacter pylori. Curr Top Microbiol Immunol.

[20] Chase CCL (2018). Enteric immunity: Happy gut, healthy animal. Vet Clin North Am Food Anim Pract.

[21] Neuman H, Koren O (2017). The Pregnancy Microbiome. Nestle Nutr Inst Workshop Ser.

[22] Asar M, Newton-Northup J, Deutscher S, Soendergaard M (2019). Ovarian cancer targeting phage for *in vivo* near-infrared optical imaging. Diagnostics (Basel).

[23] Ebrahimi S, Teimoori A, Khanbabaei H, Tabasi M Harnessing CRISPR/Cas 9 System for manipulation of DNA virus genome. Rev Med Virol. 2018: e2009.

[24] Cao B, Li Y, Yang T, Bao Q, Yang M, Mao C (2019). Bacteriophage-based biomaterials for tissue regeneration. Adv Drug Deliv Rev.

[25] Yue GG, Chan BC, Han XQ, Cheng L, Wong EC, Leung PC (2013). Immunomodulatory activities of Ganoderma sinense polysaccharides in human immune cells. Nutr Cancer.

[26] Evangelopoulos M, Parodi A, Martinez JO, Tasciotti E (2018). Trends towards biomimicry in theranostics. Nanomaterials (Basel).

[27] Blaecher C, Bauwens E, Tay A, Peters F, Dobbs S, Dobbs J (2017). A novel isolation protocol and probe-based RT-PCR for diagnosis of gastric infections with the zoonotic pathogen Helicobacter suis. Helicobacter.

[28] Gong X, Li Z, Hu Q, Zhou R, Shuang S, Dong C (2017). N,S,P co-doped carbon nanodot fabricated from waste microorganism and its application for label-free recognition of manganese(VII) and l-ascorbic acid and and logic gate operation. ACS Appl Mater Interfaces.

[29] Lee KJ, Shin SH, Lee JH, Ju EJ, Park YY, Hwang JJ (2017). A strategy for actualization of active targeting nanomedicine practically functioning in a living body. Biomaterials.

[30] Jiang P, Wang L, Hou B, Zhu J, Zhou M, Jiang J (2018). A novel HPV16 E7-affitoxin for targeted therapy of HPV16-induced human cervical cancer. Theranostics.

[31] Bateman A, Eddy SR, Mesyanzhinov VV (1997). A member of the immunoglobulin superfamily in bacteriophage T4. Virus Genes.

[32] Gamkrelidze M, Dabrowska K (2014). T4 bacteriophage as a phage display platform. Arch Microbiol.

[33] Wongthida P, Diaz RM, Galivo F, Kottke T, Thompson J, Melcher A (2011). VSV oncolytic virotherapy in the B16 model depends upon intact MyD88 signaling. Mol Ther.

[34] Chavez-Arroyo A, Portnoy DA (2020). Why is Listeria monocytogenes such a potent inducer of CD8+ T-cells?. Cell Microbiol.

[35] Han W, Chen H, Zhou L, Zou H, Luo X, Sun B (2021). Polysaccharides from Ganoderma Sinense - rice bran fermentation products and their anti-tumor activities on non-small-cell lung cancer. BMC Complement Med Ther.

[36] Ubillos L, Freire T, Berriel E, Chiribao ML, Chiale C, Festari MF (2016). Trypanosoma cruzi extracts elicit protective immune response against chemically induced colon and mammary cancers. Int J Cancer.

[37] Walker K (2006). Use of bacteriophages as novel food additives. FS06 ANR.

[38] Kutter E, Sulakvelidze A (2004). Bacteriophages: Biology and applications. 1st Edition ed. Boca Raton: CRC Press.

[39] Ivanovska IL, de Pablo PJ, Ibarra B, Sgalari G, MacKintosh FC, Carrascosa JL (2004). Bacteriophage capsids: tough nanoshells with complex elastic properties. Proc Natl Acad Sci U S A.

[40] Akhverdyan VZ, Gak ER, Tokmakova IL, Stoynova NV, Yomantas YA, Mashko SV (2011). Application of the bacteriophage Mu-driven system for the integration/amplification of target genes in the chromosomes of engineered Gram-negative bacteria-mini review. Appl Microbiol Biotechnol.

[41] Simpson AA, Tao Y, Leiman PG, Badasso MO, He Y, Jardine PJ (2000). Structure of the bacteriophage phi29 DNA packaging motor. Nature.

[42] Jamal M, Bukhari S, Andleeb S, Ali M, Raza S, Nawaz MA (2018). Bacteriophages: an overview of the control strategies against multiple bacterial infections in different fields. J Basic Microbiol.

[43] Verheust C, Pauwels K, Mahillon J, Helinski DR, Herman P (2010). Contained use of bacteriophages: risk assessment and biosafety recommendations. Applied biosafety.

[44] Zaman G, Smetsers A, Kaan A, Schoenmakers J, Konings R (1991). Regulation of expression of the genome of bacteriophage M13. Gene V protein regulated translation of the mRNAs encoded by genes I, III, V and X. Biochim Biophys Acta.

[45] Rahbarnia L, Farajnia S, Babaei H, Majidi J, Veisi K, Ahmadzadeh V (2017). Evolution of phage display technology: from discovery to application. J Drug Target.

[46] Deng X, Wang L, You X, Dai P, Zeng Y (2018). Advances in the T7 phage display system (Review). Mol Med Rep.

[47] Niemann J, Kuhnel F (2017). Oncolytic viruses: adenoviruses. Virus Genes.

[48] Barber GN (2004). Vesicular stomatitis virus as an oncolytic vector. Viral Immunol.

[49] Allen C, Vongpunsawad S, Nakamura T, James CD, Schroeder M, Cattaneo R (2006). Retargeted oncolytic measles strains entering via the EGFRvIII receptor maintain significant antitumor activity against gliomas with increased tumor specificity. Cancer Res.

[50] Kaur B, Chiocca EA, Cripe TP (2012). Oncolytic HSV-1 virotherapy: clinical experience and opportunities for progress. Curr Pharm Biotechnol.

[51] Rojas JJ, Thorne SH (2012). Theranostic potential of oncolytic vaccinia virus. Theranostics.

[52] Toth K, Wold WS (2010). Increasing the efficacy of oncolytic adenovirus vectors. Viruses.

[53] Jiang K, Song C, Kong L, Hu L, Lin G, Ye T (2018). Recombinant oncolytic Newcastle disease virus displays antitumor activities in anaplastic thyroid cancer cells. BMC Cancer.

[54] Sarotra P, Medhi B (2016). Use of bacteria in cancer therapy. Recent Results Cancer Res.

[55] Patyar S, Joshi R, Byrav DS, Prakash A, Medhi B, Das BK (2010). Bacteria in cancer therapy: a novel experimental strategy. J Biomed Sci.

[56] Zhou S, Gravekamp C, Bermudes D, Liu K (2018). Tumour-targeting bacteria engineered to fight cancer. Nat Rev Cancer.

[57] Kang SR, Jo EJ, Nguyen VH, Zhang Y, Yoon HS, Pyo A (2020). Imaging of tumor colonization by Escherichia coli using (18)F-FDS PET. Theranostics.

[58] Allemailem KS (2021). Innovative approaches of engineering tumor-targeting bacteria with different therapeutic payloads to fight cancer: A smart strategy of disease management. Int J Nanomedicine.

[59] Latge JP (2007). The cell wall: a carbohydrate armour for the fungal cell. Mol Microbiol.

[60] Erwig LP, Gow NA (2016). Interactions of fungal pathogens with phagocytes. Nat Rev Microbiol.

[61] Levitz SM (2010). Innate recognition of fungal cell walls. PLoS Pathog.

[62] Dambuza IM, Levitz SM, Netea MG, Brown GD (2017). Fungal recognition and host defense mechanisms. Microbiol Spectr.

[63] Olsson AK, Cedervall J (2018). The pro-inflammatory role of platelets in cancer. Platelets.

[64] Zappavigna S, Cossu AM, Grimaldi A, Bocchetti M, Ferraro GA, Nicoletti GF (2020). Anti-Inflammatory Drugs as Anticancer Agents. Int J Mol Sci.

[65] Sacks D, Sher A (2002). Evasion of innate immunity by parasitic protozoa. Nat Immunol.

[66] Norris KA, Bradt B, Cooper NR, So M (1991). Characterization of a Trypanosoma cruzi C3 binding protein with functional and genetic similarities to the human complement regulatory protein, decay-accelerating factor. J Immunol.

[67] Norris KA (1998). Stable transfection of Trypanosoma cruzi epimastigotes with the trypomastigote-specific complement regulatory protein cDNA confers complement resistance. Infect Immun.

[68] McConville MJ, Turco SJ, Ferguson MA, Sacks DL (1992). Developmental modification of lipophosphoglycan during the differentiation of Leishmania major promastigotes to an infectious stage. EMBO J.

[69] Raper J, Portela MP, Lugli E, Frevert U, Tomlinson S (2001). Trypanosome lytic factors: novel mediators of human innate immunity. Curr Opin Microbiol.

[70] Zuma AA, Dos Santos Barrias E, de Souza W (2021). Basic Biology of Trypanosoma cruzi. Curr Pharm Des.

[71] Agerbaek MO, Bang-Christensen S, Salanti A (2019). Fighting Cancer Using an Oncofetal Glycosaminoglycan-Binding Protein from Malaria Parasites. Trends Parasitol.

[72] Sibley LD, Andrews NW (2000). Cell invasion by un-palatable parasites. Traffic.

[73] Urban BC, Ferguson DJ, Pain A, Willcox N, Plebanski M, Austyn JM (1999). Plasmodium falciparum-infected erythrocytes modulate the maturation of dendritic cells. Nature.

[74] Tretina K, Gotia HT, Mann DJ, Silva JC (2015). Theileria-transformed bovine leukocytes have cancer hallmarks. Trends Parasitol.

[75] Su Z, Jia X, Fan Y, Zhao F, Qiao Y (2022). Progress of Research on the Relationship between Lung Microbiome and Lung Cancer. Zhongguo Fei Ai Za Zhi.

[76] Tress B, Dorn ES, Suchodolski JS, Nisar T, Ravindran P, Weber K (2017). Bacterial microbiome of the nose of healthy dogs and dogs with nasal disease. PLoS One.

[77] Gong H, Shi Y, Xiao X, Cao P, Wu C, Tao L (2017). Alterations of microbiota structure in the larynx relevant to laryngeal carcinoma. Sci Rep.

[78] Mao Q, Jiang F, Yin R, Wang J, Xia W, Dong G (2018). Interplay between the lung microbiome and lung cancer. Cancer Lett.

[79] Gholizadeh P, Eslami H, Yousefi M, Asgharzadeh M, Aghazadeh M, Kafil HS (2016). Role of oral microbiome on oral cancers, a review. Biomed Pharmacother.

[80] Raoult D (2017). Is there a link between urinary microbiota and bladder cancer?. Eur J Epidemiol.

[81] Bajic P, Wolfe AJ, Gupta GN (2019). The urinary microbiome: Implications in bladder cancer pathogenesis and therapeutics. Urology.

[82] Kwasniewski W, Wolun-Cholewa M, Kotarski J, Warchol W, Kuzma D, Kwasniewska A (2018). Microbiota dysbiosis is associated with HPV-induced cervical carcinogenesis. Oncol Lett.

[83] Liss MA, White JR, Goros M, Gelfond J, Leach R, Johnson-Pais T (2018). Metabolic biosynthesis pathways identified from fecal microbiome associated with prostate cancer. Eur Urol.

[84] Hu L, Lin Z, Wu Y, Dong J, Zhao B, Cheng Y (2016). Comprehensive profiling of EBV gene expression in nasopharyngeal carcinoma through paired-end transcriptome sequencing. Front Med.

[85] Ringelhan M, McKeating JA, Protzer U (2017). Viral hepatitis and liver cancer. Philos Trans R Soc Lond B Biol Sci.

[86] Taberna M, Mena M, Pavon MA, Alemany L, Gillison ML, Mesia R (2017). Human papillomavirus-related oropharyngeal cancer. Ann Oncol.

[87] Burger EA, Kim JJ, Sy S, Castle PE (2017). Age of acquiring causal human papillomavirus (HPV) infections: Leveraging simulation models to explore the natural history of HPV-induced cervical cancer. Clin Infect Dis.

[88] Murphy EL (2016). Infection with human T-lymphotropic virus types-1 and -2 (HTLV-1 and -2): Implications for blood transfusion safety. Transfus Clin Biol.

[89] Gao L, Michel A, Weck MN, Arndt V, Pawlita M, Brenner H (2009). Helicobacter pylori infection and gastric cancer risk: evaluation of 15 H. pylori proteins determined by novel multiplex serology. Cancer Res.

[90] Mima K, Ogino S, Nakagawa S, Sawayama H, Kinoshita K, Krashima R (2017). The role of intestinal bacteria in the development and progression of gastrointestinal tract neoplasms. Surg Oncol.

[91] Lee SH, Sung JY, Yong D, Chun J, Kim SY, Song JH (2016). Characterization of microbiome in bronchoalveolar lavage fluid of patients with lung cancer comparing with benign mass like lesions. Lung Cancer.

[92] Kew MC (2013). Aflatoxins as a cause of hepatocellular carcinoma. J Gastrointestin Liver Dis.

[93] Kim TS, Pak JH, Kim JB, Bahk YY (2016). Clonorchis sinensis, an oriental liver fluke, as a human biological agent of cholangiocarcinoma: a brief review. BMB Rep.

[94] Veeranarayanan S, Azam AH, Kiga K, Watanabe S, Cui L (2021). Bacteriophages as solid tumor theragnostic agents. Int J Mol Sci.

[95] Gorski A, Dabrowska K, Switala-Jeleń K, Nowaczyk M, Weber-Dabrowska B, Boratynski J (2003). New insights into the possible role of bacteriophages in host defense and disease. Medical Immunology.

[96] Marongiu L, Landry JJM, Rausch T, Abba ML, Delecluse S, Delecluse HJ (2021). Metagenomic analysis of primary colorectal carcinomas and their metastases identifies potential microbial risk factors. Mol Oncol.

[97] Pajtasz-Piasecka E, Rossowska J, Dus D, Weber-Dabrowska B, Zablocka A, Gorski A (2008). Bacteriophages support anti-tumor response initiated by DC-based vaccine against murine transplantable colon carcinoma. Immunol Lett.

[98] Gorski A, Wazna E, Dabrowska BW, Dabrowska K, Switala-Jelen K, Miedzybrodzki R (2006). Bacteriophage translocation. FEMS Immunol Med Microbiol.

[99] Sinkovics JG, Horvath JC (2008). Natural and genetically engineered viral agents for oncolysis and gene therapy of human cancers. Arch Immunol Ther Exp (Warsz).

[100] Gao R, Zhu Y, Kong C, Xia K, Li H, Zhu Y (2021). Alterations, interactions, and diagnostic potential of gut bacteria and viruses in colorectal cancer. Front Cell Infect Microbiol.

[101] Chaurushiya MS, Weitzman MD (2009). Viral manipulation of DNA repair and cell cycle checkpoints. DNA Repair (Amst).

[102] Akram N, Imran M, Noreen M, Ahmed F, Atif M, Fatima Z (2017). Oncogenic role of tumor viruses in humans. Viral Immunol.

[103] Read SA, Douglas MW (2014). Virus induced inflammation and cancer development. Cancer Lett.

[104] Kanda T, Goto T, Hirotsu Y, Moriyama M, Omata M (2019). Molecular mechanisms driving progression of liver cirrhosis towards hepatocellular carcinoma in chronic Hepatitis B and C infections: A review. Int J Mol Sci.

[105] Mesri EA, Cesarman E, Boshoff C (2010). Kaposi's sarcoma and its associated herpesvirus. Nat Rev Cancer.

[106] Erdman SE, Poutahidis T (2015). Gut bacteria and cancer. Biochim Biophys Acta.

[107] Rubinstein MR, Wang X, Liu W, Hao Y, Cai G, Han YW (2013). Fusobacterium nucleatum promotes colorectal carcinogenesis by modulating E-cadherin/beta-catenin signaling via its FadA adhesin. Cell Host Microbe.

[108] Nosho K, Sukawa Y, Adachi Y, Ito M, Mitsuhashi K, Kurihara H (2016). Association of Fusobacterium nucleatum with immunity and molecular alterations in colorectal cancer. World J Gastroenterol.

[109] Ramirez-Garcia A, Rementeria A, Aguirre-Urizar JM, Moragues MD, Antoran A, Pellon A (2016). Candida albicans and cancer: Can this yeast induce cancer development or progression?. Crit Rev Microbiol.

[110] Singh N, Gurav A, Sivaprakasam S, Brady E, Padia R, Shi H (2014). Activation of Gpr109a, receptor for niacin and the commensal metabolite butyrate, suppresses colonic inflammation and carcinogenesis. Immunity.

[111] Aykut B, Pushalkar S, Chen R, Li Q, Abengozar R, Kim JI (2019). The fungal mycobiome promotes pancreatic oncogenesis via activation of MBL. Nature.

[112] Daher D, Shaghlil A, Sobh E, Hamie M, Hassan ME, Moumneh MB (2021). Comprehensive overview of Toxoplasma gondii-Induced and associated diseases. Pathogens.

[113] Burki TK (2018). Phage display pioneers awarded Nobel Prize. Lancet Gastroenterol Hepatol.

[114] Weissleder R (2006). Molecular imaging in cancer. Science.

[115] Egami Y, Narushima Y, Ohshima M, Yoshida A, Yoneta N, Masaki Y (2018). Human recombinant Fab fragment from combinatorial libraries of a B-cell lymphoma patient recognizes core protein of chondroitin sulphate proteoglycan 4. J Biochem.

[116] Wang CH, Weng CH, Che YJ, Wang K, Lee GB (2015). Cancer cell-specific oligopeptides selected by an integrated microfluidic system from a phage display library for ovarian cancer diagnosis. Theranostics.

[117] El-Sayed A, Bernhard W, Barreto K, Gonzalez C, Hill W, Pastushok L (2018). Evaluation of antibody fragment properties for near-infrared fluorescence imaging of HER3-positive cancer xenografts. Theranostics.

[118] Hou J, Shen J, Zhao N, Yang CT, Thierry B, Zhou X (2021). Detection of a single circulating tumor cell using a genetically engineered antibody-like phage nanofiber probe. Mater Today Adv.

[119] Saw PE, Song EW (2019). Phage display screening of therapeutic peptide for cancer targeting and therapy. Protein Cell.

[120] Zhou X, Cao P, Zhu Y, Lu W, Gu N, Mao C (2015). Phage-mediated counting by the naked eye of miRNA molecules at attomolar concentrations in a Petri dish. Nat Mater.

[121] Pan P, Wang Y, Zhu Y, Gao X, Ju Z, Qiu P (2015). Nontoxic virus nanofibers improve the detection sensitivity for the anti-p53 antibody, a biomarker in cancer patients. Nano Res.

[122] Li Y, Qu X, Cao B, Yang T, Bao Q, Yue H (2020). Selectively Suppressing Tumor Angiogenesis for Targeted Breast Cancer Therapy by Genetically Engineered Phage. Adv Mater.

[123] Bedi D, Gillespie JW, Petrenko VA Jr, Ebner A, Leitner M, Hinterdorfer P (2013). Targeted delivery of siRNA into breast cancer cells via phage fusion proteins. Mol Pharm.

[124] Frenzel A, Schirrmann T, Hust M (2016). Phage display-derived human antibodies in clinical development and therapy. MAbs.

[125] Younes A, Vose JM, Zelenetz AD, Smith MR, Burris HA, Ansell SM (2010). A Phase 1b/2 trial of mapatumumab in patients with relapsed/refractory non-Hodgkin's lymphoma. Br J Cancer.

[126] Trarbach T, Moehler M, Heinemann V, Kohne CH, Przyborek M, Schulz C (2010). Phase II trial of mapatumumab, a fully human agonistic monoclonal antibody that targets and activates the tumour necrosis factor apoptosis-inducing ligand receptor-1 (TRAIL-R1), in patients with refractory colorectal cancer. Br J Cancer.

[127] Camidge DR, Herbst RS, Gordon MS, Eckhardt SG, Kurzrock R, Durbin B (2010). A phase I safety and pharmacokinetic study of the death receptor 5 agonistic antibody PRO95780 in patients with advanced malignancies. Clin Cancer Res.

[128] Goetz C, Gromeier M (2010). Preparing an oncolytic poliovirus recombinant for clinical application against glioblastoma multiforme. Cytokine Growth Factor Rev.

[129] Kirn DH, Thorne SH (2009). Targeted and armed oncolytic poxviruses: a novel multi-mechanistic therapeutic class for cancer. Nat Rev Cancer.

[130] Barber GN (2005). VSV-tumor selective replication and protein translation. Oncogene.

[131] Harrington KJ, Vile RG, Melcher A, Chester J, Pandha HS (2010). Clinical trials with oncolytic reovirus: moving beyond phase I into combinations with standard therapeutics. Cytokine Growth Factor Rev.

[132] David RM, Doherty AT (2017). Viral vectors: The road to reducing genotoxicity. Toxicol Sci.

[133] Rivera AA, Wang M, Suzuki K, Uil TG, Krasnykh V, Curiel DT (2004). Mode of transgene expression after fusion to early or late viral genes of a conditionally replicating adenovirus via an optimized internal ribosome entry site *in vitro* and *in vivo*. Virology.

[134] Funston GM, Kallioinen SE, de Felipe P, Ryan MD, Iggo RD (2008). Expression of heterologous genes in oncolytic adenoviruses using picornaviral 2A sequences that trigger ribosome skipping. J Gen Virol.

[135] Robertson MG, Eidenschink BB, Iguchi E, Zakharkin SO, LaRocca CJ, Tolosa EJ (2021). Cancer imaging and therapy utilizing a novel NIS-expressing adenovirus: The role of adenovirus death protein deletion. Mol Ther Oncolytics.

[136] Veerapong J, Bickenbach KA, Shao MY, Smith KD, Posner MC, Roizman B (2007). Systemic delivery of (gamma1)34.5-deleted herpes simplex virus-1 selectively targets and treats distant human xenograft tumors that express high MEK activity. Cancer Res.

[137] Msaouel P, Iankov ID, Allen C, Morris JC, von Messling V, Cattaneo R (2009). Engineered measles virus as a novel oncolytic therapy against prostate cancer. Prostate.

[138] Xu P, Wang X, Li Y, Qiu J (2019). Establishment of a Parvovirus B19 NS1-expressing recombinant adenoviral vector for killing megakaryocytic leukemia cells. Viruses.

[139] Haddad D, Chen NG, Zhang Q, Chen CH, Yu YA, Gonzalez L (2011). Insertion of the human sodium iodide symporter to facilitate deep tissue imaging does not alter oncolytic or replication capability of a novel vaccinia virus. J Transl Med.

[140] Moussavi M, Fazli L, Tearle H, Guo Y, Cox M, Bell J (2010). Oncolysis of prostate cancers induced by vesicular stomatitis virus in PTEN knockout mice. Cancer Res.

[141] Ebert O, Shinozaki K, Huang TG, Savontaus MJ, Garcia-Sastre A, Woo SL (2003). Oncolytic vesicular stomatitis virus for treatment of orthotopic hepatocellular carcinoma in immune-competent rats. Cancer Res.

[142] Goel A, Carlson SK, Classic KL, Greiner S, Naik S, Power AT (2007). Radioiodide imaging and radiovirotherapy of multiple myeloma using VSV(Delta51)-NIS, an attenuated vesicular stomatitis virus encoding the sodium iodide symporter gene. Blood.

[143] Atasheva S, Emerson CC, Yao J, Young C, Stewart PL, Shayakhmetov DM (2020). Systemic cancer therapy with engineered adenovirus that evades innate immunity. Sci Transl Med.

[144] Gromeier M, Nair SK (2018). Recombinant Poliovirus for Cancer Immunotherapy. Annu Rev Med.

[145] Guo ZS, Lu B, Guo Z, Giehl E, Feist M, Dai E (2019). Vaccinia virus-mediated cancer immunotherapy: cancer vaccines and oncolytics. J Immunother Cancer.

[146] Yang X, Huang B, Deng L, Hu Z (2018). Progress in gene therapy using oncolytic vaccinia virus as vectors. J Cancer Res Clin Oncol.

[147] Goodman B, Gardner H (2018). The microbiome and cancer. J Pathol.

[148] Duong MT, Qin Y, You SH, Min JJ (2019). Bacteria-cancer interactions: bacteria-based cancer therapy. Exp Mol Med.

[149] Raza A, Kohila V, Ghosh SS (2015). Redesigned Escherichia coli cytosine deaminase: a new facet of suicide gene therapy. J Gene Med.

[150] Luo X, Li Z, Lin S, Le T, Ittensohn M, Bermudes D (2001). Antitumor effect of VNP20009, an attenuated Salmonella, in murine tumor models. Oncol Res.

[151] Liu L, Zhang J, Gu M, Li G, Ni J, Fan M (2020). Antitumor effect of cycle inhibiting factor expression in colon cancer via Salmonella VNP20009. Anticancer Agents Med Chem.

[152] Flickinger JC Jr, Rodeck U, Snook AE (2018). Listeria monocytogenes as a vector for cancer immunotherapy: Current understanding and progress. Vaccines (Basel).

[153] Gardlik R, Fruehauf JH (2010). Bacterial vectors and delivery systems in cancer therapy. IDrugs.

[154] Xiu D, Cheng M, Zhang W, Ma X, Liu L (2020). Pseudomonas aeruginosa-mannose-sensitive hemagglutinin inhibits chemical-induced skin cancer through suppressing hedgehog signaling. Exp Biol Med (Maywood).

[155] Wong JH, Sze SCW, Ng TB, Cheung RCF, Tam C, Zhang KY (2018). Apoptosis and anti-cancer drug discovery: The power of medicinal fungi and plants. Curr Med Chem.

[156] Hirahara N, Edamatsu T, Fujieda A, Fujioka M, Wada T, Tajima Y (2013). Protein-bound polysaccharide-K induces apoptosis via mitochondria and p38 mitogen-activated protein kinase-dependent pathways in HL-60 promyelomonocytic leukemia cells. Oncol Rep.

[157] Dan X, Liu W, Wong JH, Ng TB (2016). A Ribonuclease Isolated from Wild Ganoderma Lucidum Suppressed Autophagy and Triggered Apoptosis in Colorectal Cancer Cells. Front Pharmacol.

[158] Liao Y, Ling J, Zhang G, Liu F, Tao S, Han Z (2015). Cordycepin induces cell cycle arrest and apoptosis by inducing DNA damage and up-regulation of p53 in Leukemia cells. Cell Cycle.

[159] He JB, Tao J, Miao XS, Bu W, Zhang S, Dong ZJ (2015). Seven new drimane-type sesquiterpenoids from cultures of fungus Laetiporus sulphureus. Fitoterapia.

[160] Sun Y, Hu X, Li W (2017). Antioxidant, antitumor and immunostimulatory activities of the polypeptide from Pleurotus eryngii mycelium. Int J Biol Macromol.

[161] Ma L, Chen H, Dong P, Lu X (2013). Anti-inflammatory and anticancer activities of extracts and compounds from the mushroom Inonotus obliquus. Food Chem.

[162] Park JH, Ryu CS, Kim HN, Na YJ, Park HJ, Kim H (2004). A sialic acid-specific lectin from the mushroom Paecilomyces Japonica that exhibits hemagglutination activity and cytotoxicity. Protein Pept Lett.

[163] Quang DN, Lam DM, Hanh NT, Que do D (2013). Cytotoxic constituents from the fungus Daldinia concentrica (Xylariaceae). Nat Prod Res.

[164] Linh DT, Hien BT, Que do D, Lam DM, Arnold N, Schmidt J (2014). Cytotoxic constituents from the Vietnamese fungus Xylaria schweinitzii. Nat Prod Commun.

[165] Li Y, Zhang G, Ng TB, Wang H (2010). A novel lectin with antiproliferative and HIV-1 reverse transcriptase inhibitory activities from dried fruiting bodies of the monkey head mushroom Hericium erinaceum. J Biomed Biotechnol.

[166] Zhao S, Zhao Y, Li S, Zhao J, Zhang G, Wang H (2010). A novel lectin with highly potent antiproliferative and HIV-1 reverse transcriptase inhibitory activities from the edible wild mushroom Russula delica. Glycoconj J.

[167] Zhang G, Sun J, Wang H, Ng TB (2010). First isolation and characterization of a novel lectin with potent antitumor activity from a Russula mushroom. Phytomedicine.

[168] Li M, Zhang G, Wang H, Ng T (2010). Purification and characterization of a laccase from the edible wild mushroom Tricholoma mongolicum. J Microbiol Biotechnol.

[169] Vaz JA, Heleno SA, Martins A, Almeida GM, Vasconcelos MH, Ferreira IC (2010). Wild mushrooms Clitocybe alexandri and Lepista inversa: *in vitro* antioxidant activity and growth inhibition of human tumour cell lines. Food Chem Toxicol.

[170] Arora D, Kumar A, Gupta P, Chashoo G, Jaglan S (2017). Preparation, characterization and cytotoxic evaluation of bovine serum albumin nanoparticles encapsulating 5-methylmellein: A secondary metabolite isolated from Xylaria psidii. Bioorg Med Chem Lett.

[171] Noppawan S, Mongkolthanaruk W, Suwannasai N, Senawong T, Moontragoon P, Boonmak J (2020). Chemical constituents and cytotoxic activity from the wood-decaying fungus Xylaria sp. SWUF08-37. Nat Prod Res.

[172] Zhang L, Khoo C, Koyyalamudi SR, Pedro Nd, Reddy N (2017). Antioxidant, anti-inflammatory and anticancer activities of ethanol soluble organics from water extracts of selected medicinal herbs and their relation with flavonoid and phenolic contents. Pharmacologia.

[173] Lin YS, Lin YY, Yang YH, Lin CL, Kuan FC, Lu CN (2018). Antrodia cinnamomea extract inhibits the proliferation of tamoxifen-resistant breast cancer cells through apoptosis and skp2/microRNAs pathway. BMC Complement Altern Med.

[174] Zhang P, Li K, Yang G, Xia C, Polston JE, Li G (2017). Cytotoxic protein from the mushroom Coprinus comatus possesses a unique mode for glycan binding and specificity. Proc Natl Acad Sci U S A.

[175] Gu YH, Leonard J (2006). *In vitro* effects on proliferation, apoptosis and colony inhibition in ER-dependent and ER-independent human breast cancer cells by selected mushroom species. Oncol Rep.

[176] Wong JH, Ng TB, Chan HHL, Liu Q, Man GCW, Zhang CZ (2020). Mushroom extracts and compounds with suppressive action on breast cancer: evidence from studies using cultured cancer cells, tumor-bearing animals, and clinical trials. Appl Microbiol Biotechnol.

[177] Wang D, Sun SQ, Wu WZ, Yang SL, Tan JM (2014). Characterization of a water-soluble polysaccharide from Boletus edulis and its antitumor and immunomodulatory activities on renal cancer in mice. Carbohydr Polym.

[178] Torello CO, Souza-Queiroz J, Queiroz ML (2012). beta-1,3-Glucan given orally modulates immunomyelopoietic activity and enhances the resistance of tumour-bearing mice. Clin Exp Pharmacol Physiol.

[179] Steimbach L, Borgmann AV, Gomar GG, Hoffmann LV, Rutckeviski R, de Andrade DP (2021). Fungal beta-glucans as adjuvants for treating cancer patients - A systematic review of clinical trials. Clin Nutr.

[180] Alonso EN, Ferronato MJ, Gandini NA, Fermento ME, Obiol DJ, Lopez Romero A (2017). Antitumoral effects of D-Fraction from Grifola frondosa (Maitake) mushroom in breast cancer. Nutr Cancer.

[181] Liu X, Wu X, Ma Y, Zhang W, Hu L, Feng X (2017). Endophytic fungi from mangrove inhibit lung cancer cell growth and angiogenesis *in vitro*. Oncol Rep.

[182] Wu HT, Lu FH, Su YC, Ou HY, Hung HC, Wu JS (2014). *In vivo* and *in vitro* anti-tumor effects of fungal extracts. Molecules.

[183] Callejas BE, Martinez-Saucedo D, Terrazas LI (2018). Parasites as negative regulators of cancer. Biosci Rep.

[184] Baindara P, Mandal SM (2020). Bacteria and bacterial anticancer agents as a promising alternative for cancer therapeutics. Biochimie.

[185] Hunter CA, Subauste CS, Van Cleave VH, Remington JS (1994). Production of gamma interferon by natural killer cells from Toxoplasma gondii-infected SCID mice: regulation by interleukin-10, interleukin-12, and tumor necrosis factor alpha. Infect Immun.

[186] Shapira S, Speirs K, Gerstein A, Caamano J, Hunter CA (2002). Suppression of NF-kappaB activation by infection with Toxoplasma gondii. J Infect Dis.

[187] Xie YH, Gao QY, Cai GX, Sun XM, Sun XM, Zou TH (2017). Fecal Clostridium symbiosum for noninvasive detection of early and advanced colorectal cancer: test and validation studies. EBioMedicine.

[188] Streby KA, Currier MA, Triplet M, Ott K, Dishman DJ, Vaughan MR (2019). First-in-human intravenous Seprehvir in young cancer patients: A phase 1 clinical trial. Mol Ther.

[189] Newgard CB (2017). Metabolomics and Metabolic Diseases: Where Do We Stand?. Cell Metab.

[190] Watts GS, Youens-Clark K, Slepian MJ, Wolk DM, Oshiro MM, Metzger GS (2017). 16S rRNA gene sequencing on a benchtop sequencer: accuracy for identification of clinically important bacteria. J Appl Microbiol.

[191] Bradley P, den Bakker HC, Rocha EPC, McVean G, Iqbal Z (2019). Ultrafast search of all deposited bacterial and viral genomic data. Nat Biotechnol.

[192] Xue X, Wang B, Du W, Zhang C, Song Y, Cai Y (2016). Generation of affibody molecules specific for HPV16 E7 recognition. Oncotarget.

[193] Gorski A, Weber-Dabrowska B (2005). The potential role of endogenous bacteriophages in controlling invading pathogens. Cell Mol Life Sci.

[194] Thirukkumaran CM, Shi ZQ, Luider J, Kopciuk K, Gao H, Bahlis N (2012). Reovirus as a viable therapeutic option for the treatment of multiple myeloma. Clin Cancer Res.

[195] Qiu J, Zhang H, Wang Z (2018). Auricularia auriculajudae polysaccharide-cisplatin complexes conjugated with folic acid as new tumor targeting agents. Int J Biol Macromol.

[196] Konagai A, Yoshimura K, Hazama S, Yamamoto N, Aoki K, Ueno T (2017). Correlation Between NKG2DL Expression and Antitumor Effect of Protein-bound Polysaccharide-K in Tumor-bearing Mouse Models. Anticancer Res.

[197] Krishnamoorthy D, Sankaran M (2016). Modulatory effect of Pleurotus ostreatus on oxidant/antioxidant status in 7, 12-dimethylbenz (a) anthracene induced mammary carcinoma in experimental rats-A dose-response study. J Cancer Res Ther.

[198] Motamedi M, Arab S, Moazzeni SM, Khamis Abadi M, Hadjati J (2009). Improvement of a dendritic cell-based therapeutic cancer vaccine with components of Toxoplasma gondii. Clin Vaccine Immunol.

